# Neotropical *Nilothauma* Kieffer, 1921 (Diptera, Chironomidae): Key, eleven new species, re-descriptions, new combination and new records

**DOI:** 10.3897/zookeys.1033.60686

**Published:** 2021-04-22

**Authors:** Luiz Carlos Pinho, Trond Andersen

**Affiliations:** 1 Laboratory of Systematics of Diptera, Department of Ecology and Zoology, Federal University of Santa Catarina, Campus Trindade, CEP 88040-900, Florianópolis, Brazil Federal University of Santa Catarina Florianópolis Brazil; 2 Department of Natural History, University Museum of Bergen, University of Bergen, P.O. Box 7800, NO-5020, Bergen, Norway University of Bergen Bergen Norway

**Keywords:** Brazil, key, Mexico, Neotropical Region, new combination, new records, new species, taxonomy

## Abstract

Nine new species of *Nilothauma* Kieffer, *N. hamadae***sp. nov.**, *N. jupau***sp. nov.**, *N. karitiana***sp. nov.**, *N. leccii***sp. nov.**, *N. marianoi***sp. nov.**, *N. mateusi***sp. nov.**, *N. txukuyana***sp. nov.**, *N. werekena***sp. nov.** and *N. yekwana***sp. nov.** are described and figured, based on adult males collected in Brazil and *N. maya***sp. nov.** on an adult male from Mexico; *N. terena***sp. nov.** is described as male, pupa and larva based on a reared specimen from Brazil. *Nilothauma aleta* Roback, 1960 and *N. duena* Roback, 1960 are re-described and recorded from Brazil. *Nilothauma longissimum* Mendes & Andersen, 2009 is transferred to *Beardius* Reiss & Sublette, 1985 and the diagnosis of *Nilothauma* is emended. New records of thirteen Neotropical *Nilothauma* species are given and a key to the males of all known species of *Nilothauma* is provided.

## Introduction

The genus *Nilothauma* was erected by Kieffer (1921), based on the Afrotropical *N. pictipenne* Kieffer, 1921. The Neotropical genera *Neelamia* Soponis, 1987 and *Paranilothauma* Soponis, 1987 were both placed as synonyms of *Nilothauma* by Mendes and Andersen (2009). Most males of *Nilothauma* can be recognised by having an antenna with 13 flagellomeres, low antennal ratio (except in *Nilothauma longissimum* Mendes & Andersen, 2009), fore-tibia with long spur on conical, apical scale, high venarum ratio and squama bare (Mendes and Andersen 2009). In addition, many males have median or lateral, sometimes strongly setose lobes or projections on tergite IX. The larvae of *Nilothauma* inhabit littoral and sublittoral soft sediments of lakes, streams and rivers (Epler et al. 2013).

To date, the genus comprises 52 described species that occur in all zoogeographical regions, except Antarctica (Qi et al. 2014, 2016; Niitsuma 2016; Dantas and Hamada 2017). A total of 22 species are known from the Neotropical and four from the Nearctic Regions (Andersen et al. 2016; Dantas and Hamada 2017).

After examining material collected in several localities in the Neotropical Region, mostly from the Brazilian Amazon, 24 species of *Nilothauma* were identified. Eleven of them proved to be new to science and are described below as adult males and for *N. terena* sp. nov. also as larva and pupa. The other thirteen species have their distribution range extended in the Neotropics. *Nilothauma aleta* Roback, 1960 and *N. duena* Roback, 1960, originally described from the Peruvian Amazon are re-described, based on material from Brazil and *N. longissimum* Mendes & Andersen, 2009 is transferred to *Beardius* Reiss & Sublette, 1985. A key to the males of all known species of *Nilothauma* is provided.

## Material and methods

Alcohol-preserved specimens were dissected and slide-mounted in Euparal. Morphological terminology and abbreviations follow Sæther (1980). Measurements are taken according to Epler (1988) and given as ranges, followed by the mean when more than three specimens were measured, followed by the number of specimens measured in parenthesis.

Abbreviations used in the text as follows:

**CEPA** Centro de Estudos e Pesquisas Ambientais (Centre for Environmental Studies and Research);

**EB** Estação Biológica (Biological Station);

**INPA**Instituto Nacional de Pesquisas da Amazônia (Manaus, Brazil);

**MZSP**Museu de Zoologia da Universidade de São Paulo (São Paulo, Brazil);

**PE** Parque Estadual (State Park);

**RPPN** Reserva Particular do Patrimônio Natural (Private Natural Heritage Reserve);

**UFSC** Entomological Collection of the Federal University of Santa Catarina (Florianópolis, Brazil);

**ZMBN**University Museum of Bergen (Bergen, Norway);

**ZSM**Zoologische Staatsammlung München (Munich, Germany).

Type material is deposited at INPA, MZSP, UFSC, ZMBN and ZSM, as stated in each description. Vouchers are deposited at UFSC, ZMBN and ZSM.

## Taxonomy

### *Beardius* Reiss & Sublette, 1985

#### Beardius longissimus

Taxon classificationAnimaliaDipteraChironomidae

(Mendes & Andersen, 2009) comb. nov.

3A5357FE-12A3-5AA6-AC88-AEB3C8120BB7

Nilothauma longissimum Mendes & Andersen, 2009: 26

##### Material examined.

Type material, as in Mendes and Andersen (2009).

##### Additional material.

8 males, slide-mounted: Brazil, Bahia, Camacan, RPPN Serra Bonita, Trilha Bapeba,15°20'35"S, 39°33'34"W, 4.xi.2009, light trap, A.R. Calor et al. leg. 3 males, slide-mounted, as previous, except: 15°23'32"S, 39°33'53"W, 2.xi.2009. 1 male, slide-mounted, as previous, except: 03.ii.2009. 1 male, slide-mounted, as previous, except: córrego 2, 15°23'10"S, 39°34'03"W, 819 m a.s.l., 01.viii.2008, light trap, A.R. Calor, L.S. Lecci, L.C. Pinho & R.A. Moretto leg. 1 male, slide-mounted: Brazil, São Paulo, PE Serra do Mar, Ubatuba, Picinguaba, Camburi stream, 09.ix.2006, light trap, M.R. Spies & A.E. Siegloch leg.

##### Remarks.

Pinho et al. (2013) found that the “presence of apical thin setae on inferior volsella” (character 74, state 1), i.e. a group of two, rarely three, slender simple setae at the very tip of the inferior volsella, in addition to subapical, stouter setae, is the only synapomorphy of *Beardius* Reiss & Sublette, 1985 in the adult stage. In fact, the character is shared by all species of *Beardius* and is not found elsewhere, except in *Nilothauma longissimum* Mendes & Andersen, 2009, a species that was considered to be sister to all remaining *Nilothauma* by Mendes and Andersen (2009) in a phylogenetic analysis with *Paratendipes* Kieffer and *Pseudochironomus* Malloch as outgroups. The comparatively-high antennal ratio in *N. longissimum* (AR > 1.00) is the only exception in *Nilothauma*, which generally have very low antennal ratios (AR < 0.40). Further, the venarum ratio (VR) seems to be low in *N. longissimum* compared to other *Nilothauma* species; other characters in *N. longissimum* are consistent with the current diagnosis of *Beardius*. We therefore propose the new combination and emend the diagnosis of *Nilothauma* accordingly.

##### Distribution.

The species was originally described from São Paulo State, south-eastern Brazil by Mendes and Andersen (2009); the range is now extended to Bahia State in north-eastern Brazil.

#### Nilothauma

Taxon classificationAnimaliaDipteraChironomidae

Kieffer, 1921

970EB777-ABA5-5FD1-9BAD-4EDE72E1D845

##### Emended diagnosis.

After transferring *Nilothauma longissimum* Mendes & Andersen, 2009 to *Beardius* Reiss & Sublette, 1985 (see above), adult males of *Nilothauma* become more easily separated from other genera. The diagnosis given by Mendes and Andersen (2009) has to be emended as follows: “antennal ratio generally low (AR < 0.40), one species (*N. longissimum* sp. nov.) with AR > 1.00.” should read: “antennal ratio generally low (AR < 0.40), occasionally as high as 0.82 (*N. soka* Andersen, Bello González & Hagenlund, 2016).

The discovery of the pupae of *N. terena* sp. nov. leads to the diagnosis of the pupa given by Mendes and Andersen (2009) having to be emended as follows: “Frontal setae short, not on tubercles.” should read “Frontal setae short, occasionally long and taeniate, not on tubercles.” Further, “Sternites I–VII bare; sternite VIII with central, longitudinal field of shagreen.” should read “Sternites I–VII usually bare, sternite I occasionally with extensive shagreen; sternite VIII with central, longitudinal field of shagreen.”

Based on the larva of *N. terena* sp. nov., the diagnosis of the *Nilothauma* larvae in Epler et al. (2013) should be emended as follows: “Mandible. All teeth pale;” should read: “Mandible. All teeth pale, occasionally inner teeth with somewhat darker pigmentation;” and “Mentum. Pale;” should read: “Mentum. Pale, occasionally with somewhat darker pigmentation”.

#### Nilothauma aleta

Taxon classificationAnimaliaDipteraChironomidae

Roback, 1960

9383FBE1-EA80-55DE-B427-AB89CF16F4BF

Figures 1A, B , 17B

##### Additional material.

1 male, slide-mounted: Brazil, São Paulo, São Luís do Paraitinga, PE Serra do Mar, Núcleo Santa Virgínia, trilha Poço do Pito, afluente Paraibuna, 23°20'09"S, 45°08'46"W, 15.ix.2006, light trap, M.R. Spies & A.E. Siegloch leg.

##### Diagnostic characters.

The male can be distinguished from its congeners by the combination of: tergite IX without setose dorsal lobe(s); gonostylus stout; acrostichals absent; anal point wide, covering most setae along posterior margin of tergite IX; inferior volsella slender.

##### Re-description.

**Male imago (n = 1).** Total length 3.58 mm. Wing length 2.00 mm. Total length/wing length 1.79. Wing length/length of profemur 2.25.

***Colouration*.** Head, thorax and abdomen brown; legs pale, except for ring of brown pigmentation in distal 1/2 to 2/3 of fore- and hind femora, in distal 1/3 of foretibia, in basal 1/8 of mid- and hind tibiae and in distal 1/3 of each tarsomere. Wing membrane apparently hyaline, but faint brown markings are visible when dark-field filter is applied.

***Antenna*.**AR = 0.27. Thirteenth flagellomere 197 µm long.

***Head*.** Temporal setae 7 in single row. Clypeus with 25 setae. Tentorium 123 µm long, maximum width 25 µm. Stipes not measurable. Palp segment lengths (in µm): 39, 34, 123, 147, 191. Third palpomere with 2 sensilla clavata subapically, longest 20 µm long. Fifth palpomere/third palpomere 1.55.

***Thorax*.** Dorsocentrals 12 in single row, acrostichals absent, prealars 3. Scutellum with 6 setae.

***Wing*.**VR = 1.50. Brachiolum with 1 seta, R with 13 setae, R_1_ with 18 setae, R_4+5_ with 21 setae, remaining veins bare.

***Legs*.** Spur of fore tibia 44 µm long including 15 µm long scale. Mid-tibia with 1 spur, 15 µm long; hind tibia with 2 spurs, 25 and 29 µm long. Combs of both mid- and hind tibia 20 µm long. Width at apex of fore-tibia 39 µm, of mid-tibia 34 µm, of hind tibia 44 µm. Lengths and proportions of legs as in Table 1.

**Table 1.** Lengths (in μm) and proportions of leg segments in *Nilothauma aleta* Roback, 1960, adult male (n = 1).

	**Fe**	**Ti**	**ta_1_**	**ta_2_**	**ta_3_**	**ta_4_**
p_1_	887	601	837	522	404	256
p_2_	906	690	414	227	177	118
p_3_	1034	985	699	355	305	197
	**ta_5_**	**LR**	**BV**	**SV**	**BR**	
p_1_	148	1.39	1.74	1.77	2.3	
p_2_	89	0.60	3.29	2.30	2.3	
p_3_	108	0.71	2.56	2.89	5.0	

**Figure 1.**
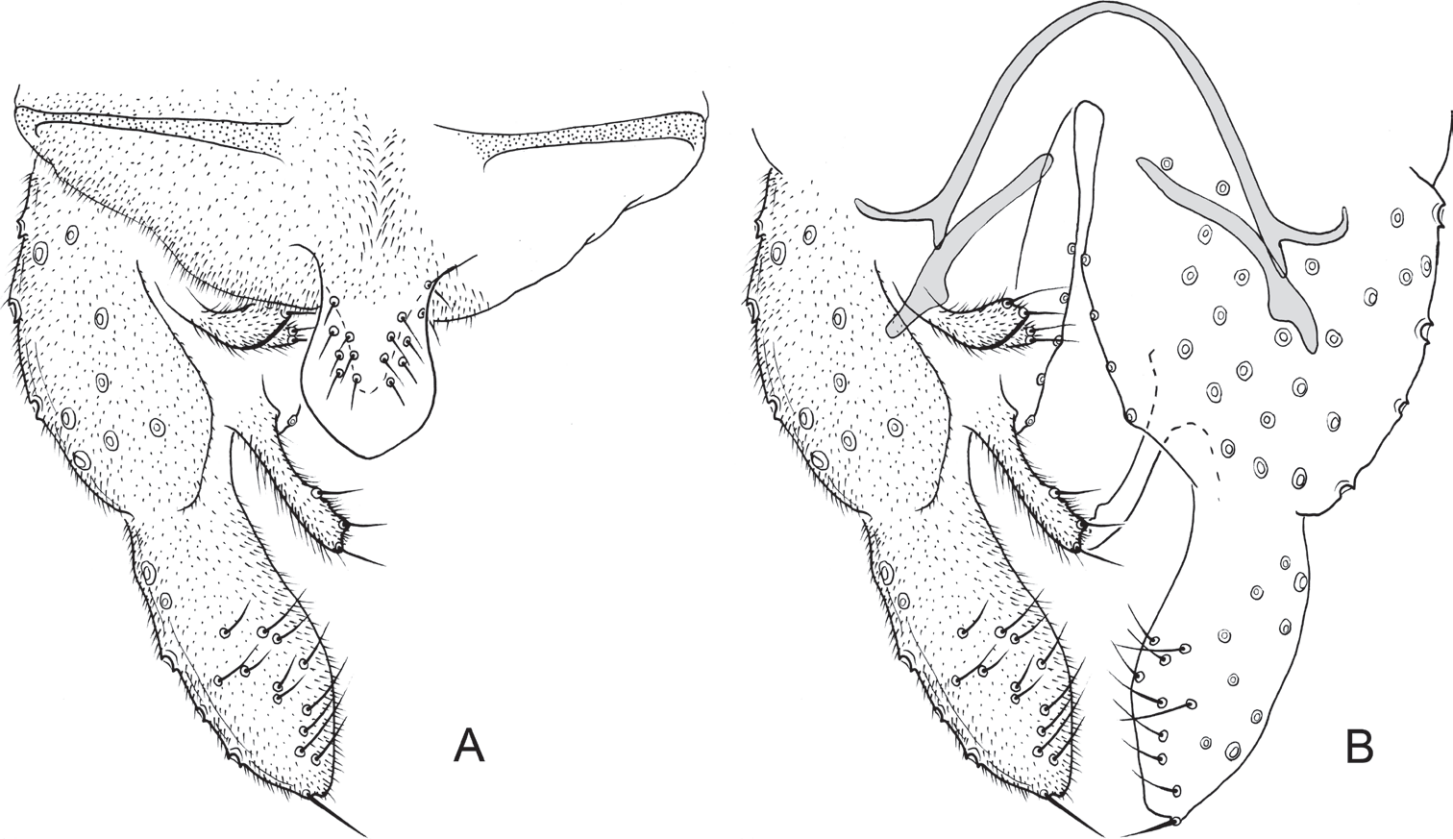
*Nilothauma aleta* Roback, 1960, adult male **A** hypopygium, dorsal view **B** hypopygium with anal point and tergite IX removed, dorsal aspect to the left and ventral aspect to the right.

***Hypopygium*** (Fig. 1A, B). Tergite IX without lobes, tapering to apex, with 22 short setae underneath anal point. Anal point lanceolate, 50 µm long, maximum width 37 µm. Tergite bands well developed. Laterosternite IX without setae. Phallapodeme 70 µm long; transverse sternapodeme 55 µm long. Gonocoxite 134 µm long. Inferior volsella straight, 52 µm long, 7 µm wide medially, with microtrichia and 8 simple setae apically. Superior volsella pediform, 17 µm long, 7 µm wide at base, covered with microtrichia and with 2 setae apically. Median volsella 7 µm long, with 2 simple setae, longest 12 µm. Gonostylus 95 µm long, straight. HR = 1.42. HV = 3.77.

##### Female adult and immatures.

Unknown.

##### Remarks.

Roback (1960) described *Nilothauma aleta* Roback, 1960 and *N. duena* Roback, 1960 from the Peruvian Amazon. In their revision of *Nilothauma*, Adam & Sæther, (1999) regarded the two species as not belonging to *Nilothauma* since they lack any projections on tergite IX and stated that they appear to belong in *Paratendipes* Kieffer. Later, Mendes and Andersen (2009) placed *Neelamia* Soponis and *Paranilothauma* Soponis as synonyms of *Nilothauma* and several new Neotropical species have been described demonstrating the large morphological variation in the genus. Mendes and Andersen (2009) emended the diagnosis of *Nilothauma* and both *N. aleta* and *N. duena* fit well into this diagnosis.

##### Distribution

**(Fig. 17B).** The species was originally described from the Department of Huánuco, in the Peruvian Amazon by Roback (1960); the range is now extended to Serra do Mar (São Paulo State), in the Brazilian Atlantic Forest.

#### Nilothauma amazonense

Taxon classificationAnimaliaDipteraChironomidae

Mendes & Andersen, 2009

77B5F05C-7FE9-5DA5-8CED-0D65696CE65D

Figure 17A

##### Additional material.

1 male, slide-mounted: Brazil, Santa Catarina, Grão Pará, Cachoeira do Amado, #27, 28°08'57"S, 49°21'17"W, 16.xi.2012–08.i.2013, Malaise trap, L.C. Pinho, M.C. Novaes & M.F. Haddad leg. 1 male, slide-mounted: Brazil, Santa Catarina, São Francisco do Sul, Distrito do Saí, 26°11'42"S, 48°43'53"W, 18.i–18.iii.2020, Malaise trap #150, small stream, L.C. Pinho et al. leg.

##### Remarks.

*Nilothauma amazonense* Mendes & Andersen, 2009 was described, based on a single male from the Amazon. The specimens of *N. amazonense* from southern Brazil, however, differ slightly from the holotype. Mendes and Andersen (2009) stated that hind ta_2_ being shorter than ta_3_ (ratio of ta_2_/ta_3_ length = 0.73) is one of the diagnostic characters of the species. However, in the specimens from southern Brazil, hind ta_2_ and ta_3_ are subequal in length (ratio of ta_2_/ta_3_ length = 0.94–0.97). Body size, measured as Total Length (TL) is also larger (TL of holotype = 1.53 mm; TL of southern populations = 2.00–2.05 mm). Similar differences in body size between Amazonian and southern Atlantic Forest populations were also found in *Beardius urupeatan* Pinho, Mendes & Andersen, 2009 [TL Amazon = 2.32–2.51, 2.38 (6); TL southern Atlantic Forest = 2.68–3.00, 2.96 (8)]. This intraspecific variation might be due to the higher temperature in the Amazon Region when compared to the localities in the southern parts of the Atlantic Forest. Populations of chironomid species inhabiting different habitats may show variation in voltinism and more rapid growth can result in smaller body size (Tokeshi 1995; Pinho et al. 2009).

##### Distribution

**(Fig. 17A).** The species was originally described from the Amazonian Region by Mendes and Andersen (2009); the range is now extended to Santa Catarina State in southern Brazil.

#### Nilothauma anamariae

Taxon classificationAnimaliaDipteraChironomidae

Dantas & Hamada, 2017

D6A5A738-7BD7-5112-B8B4-C3CD55D88C40

Figure 17D

##### Additional material.

1 male, slide-mounted: Brazil, Rondônia, Candeias do Jamari, Rio Preto, Ponte de Madeira, #01, 08°52'40"S, 63°38'02"W, 19–20.vii.2012, light trap, R. Boldrini & A.S. Fernandes leg. 1 male, slide mounted: Brazil, Mato Grosso, Ribeirão Cascalheira, Fazenda Campina Grande, Rio Suiá Miçu, 28–30.xi.2006, light trap, A.R. Calor, F.R. Silva & S. Mateus leg. 2 males, slide-mounted: Brazil, Mato Grosso, Ribeirão Cascalheira, Fazenda Campina Verde, Rio Suiá Miçu, 12°48.591'S, 52°06.925'W, 10.x.2007, light trap, L.C. Pinho, S. Mateus, L. Torati & F.R. Silva leg. 1 male, slide-mounted: Brazil, Pará State, Rurópolis, Rio Tambor, 29.x.2007, light trap, N. Hamada et al. leg.

##### Remarks.

The inferior volsella can have up to 3–4 simple, curved setae apically.

##### Distribution

**(Fig. 17D).** The species was originally described from the Rio Grande do Sul State in southern Brazil by Dantas and Hamada (2017); the range is now extended to Mato Grosso, Rondônia and Pará States in central and northern Brazil.

#### Nilothauma aripuanense

Taxon classificationAnimaliaDipteraChironomidae

Mendes & Andersen, 2009

A5B525B1-7F33-5F85-8317-C1367FB1B98A

Figure 17B

##### Additional material.

2 males, slide-mounted: Brazil, Rondônia, Candeias do Jamari, Rio Preto, Ponte de Madeira, #01, 08°52'40"S, 63°38'02"W, 19–20.vii.2012, light trap, R. Boldrini & A.S. Fernandes leg. 2 males, slide-mounted: Brazil, Rondônia, Teixeirópolis, Balneário com Cachoeira, 10°55'20"S, 62°22'34"W, 03.ix.2012, light #13, N. Hamada, R. Boldrini, A.S. Fernandes & J.M. Cavalcante leg. 1 male, slide-mounted: Brazil, Roraima, Boa Vista, Rio Cauamé, 02°52'06"N, 60°44'24"W, 9.iii.2009, light trap, L.M. Fusari leg. 1 male, slide-mounted: Brazil, Roraima, Boa Vista, BR-174, Igarapé Água Boa, 02°43'32"N, 60°48'43"W, 2014, N. Hamada leg. 1 male, slide-mounted: Brazil, Amazonas, Presidente Figueiredo, AM-240 Km 20, Balneário Sossego da Pantera, Igarapé da Onça, 02°02'31"S, 59°51'05"W, 02.vii.2008, light trap, C. Azevedo leg. 1 male, slide-mounted: Brazil, Amazonas, upper Rio Marauiá, downstream of Cachoeira Santo Antônio, surface float skimmed, 22.i.1963, E.J. Fittkau leg. (A485, ZSM).

##### Distribution

**(Fig. 17B).** The species was originally described from the Amazonas and Mato Grosso States by Mendes and Andersen (2009); the range is now extended to Roraima and Rondônia States in the Brazilian Amazon.

#### Nilothauma calori

Taxon classificationAnimaliaDipteraChironomidae

Mendes & Andersen, 2009

5B3A8429-5F51-595F-A55B-B32A6D0E65C1

Figures 2A , 16C

##### Additional material.

1 male, slide-mounted: Brazil, Amazonas, Manaus, Reserva Florestal Adolfo Ducke, Igarapé Bolívia, 02°49'15"S, 59°56'31"W, 9–12.xi.2008, Malaise trap suspensa 2, N. Hamada et al. leg. 2 males, slide-mounted: Brazil, Mato Grosso, Cuiabá, 10–11.x.1965, Brundin net, E.J. Fittkau leg. (A 580, ZSM).

##### Remarks.

**Figure 2.**
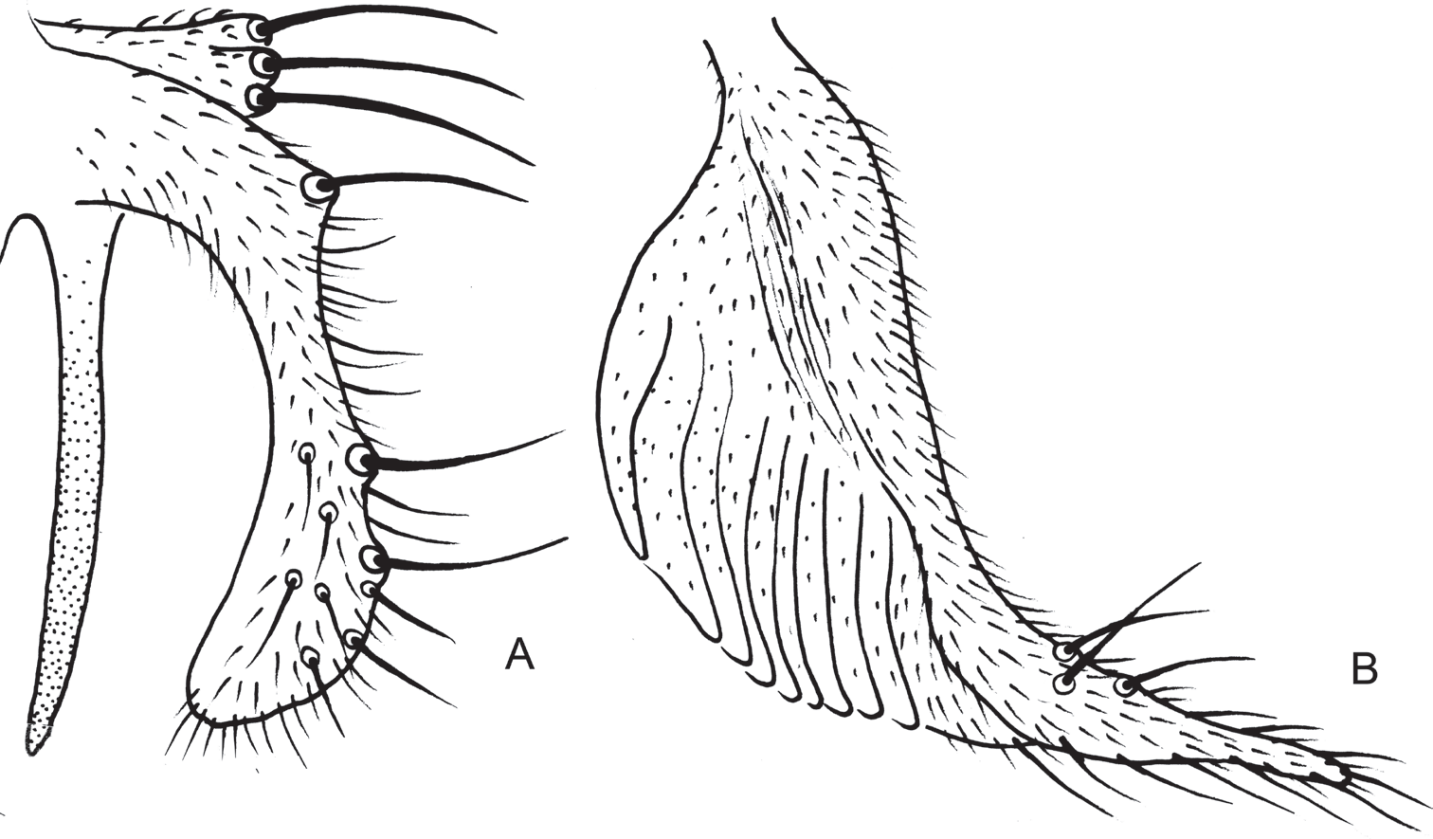
Variation in superior volsella **A** dorsolateral view of superior volsella of *Nilothauma calori* Mendes & Andersen, 2009 **B** lateral view of superior volsella of *Nilothauma complicatum* Mendes & Andersen, 2009.

Mendes and Andersen (2009) stated that the superior volsella has a “lateral strongly sclerotized, spine-like projection”. In dorsolateral view (Fig. 2A), it can be seen that this spine-like projection originates from the base of the volsella and is equally long as the volsella proper.

##### Distribution

**(Fig. 16C).** The species was originally described from Acre State by Mendes and Andersen (2009); the range is now extended to the Mato Grosso and Amazonas States.

#### Nilothauma complicatum

Taxon classificationAnimaliaDipteraChironomidae

Mendes & Andersen, 2009

D42B5AE8-49B7-58FD-A9DF-E5A7DBB0EDBA

Figures 2B , 16A

##### Additional material.

1 male, slide-mounted: Brazil, Rondônia, Candeias do Jamari, Rio Preto, Ponte de Madeira, #01, 08°52'40"S, 63°38'02"W, 19–20.vii.2012, light trap, R. Boldrini & A.S. Fernandes leg. 3 males, slide-mounted: Brazil, Mato Grosso, Nova Xavantina, Fazenda Sr. Queté, Córrego Voadeira, 14°32.187'S, 52°30.902'W, 16.x.2007, light trap, L.C. Pinho, S. Mateus, L. Torati & F.R. Silva leg. 2 males, slide-mounted, as previous, except: Córrego Cachoeira, 14°32.817'S, 52°31.395'W. 1 male, slide-mounted, as previous, except: 14°41.577'S, 52°27.203'W, 13.x.2007. 2 males, slide-mounted, as previous, except: Estrada p/ Rancho Helena, Córrego Ponte de Pedra, 14°47.908'S, 52°37.226'W, 15.x.2007. 1 male, slide-mounted, as previous, except: Córrego Voadeira, 14°41.577'S, 52°27.203'W, 13.x.2007. 1 male, slide-mounted: Brazil, Mato Grosso, Ribeirão Cascalheira, Estrada Fazenda Manaus, 1° afluente Rio Bonito, 12°57.088'S, 51°52.480'W, 08.x.2007, light trap, L.C. Pinho, S. Mateus, L. Torati & F.R. Silva leg.

##### Remarks.

Mendes and Andersen (2009) stated that the superior volsella has a “marginal row of flattened setae”. In lateral view (Fig. 2B), it can be seen that the volsella is quite wide medially with a row of lamellae apparently covered with weak microtrichia.

##### Distribution

**(Fig. 16A).** The species was originally described by Mendes and Andersen (2009), based on a single male from the Espírito Santo State; the range is now extended to the Mato Grosso and Rondônia States in central and northern Brazil.

#### Nilothauma duena

Taxon classificationAnimaliaDipteraChironomidae

Roback, 1960

A26C8588-9FBE-5FA0-83BC-BE670994ADC5

Figures 3A, B , 16C

##### Additional material.

1 male, slide-mounted: Brazil, Bahia, Camacan, Fazenda do Waldemar da farmácia, Córrego abaixo da represa de abastecimento, 15°25'16"S, 39°33'57"W, 300 m a.s.l., 05.viii.2008, light trap, A.R. Calor, L.S. Lecci, L.C. Pinho & R.A. Moretto leg. 1 male, slide-mounted: Brazil, São Paulo, Pindamonhangaba, Fazenda São Sebastião, Afluente Cedro 3, 22°50'16"S, 45°28'27"W, 18.ix.2006, light trap, M.R. Spies & A.E. Siegloch leg.

##### Diagnostic characters.

The male can be distinguished from its congeners by the combination of: tergite IX without setose dorsal lobe(s); anal point slightly spatulate; wing unmarked; abdominal tergites I–VIII with basal half light brown, distal half pale; gonostylus and inferior volsella stout.

**Figure 3.**
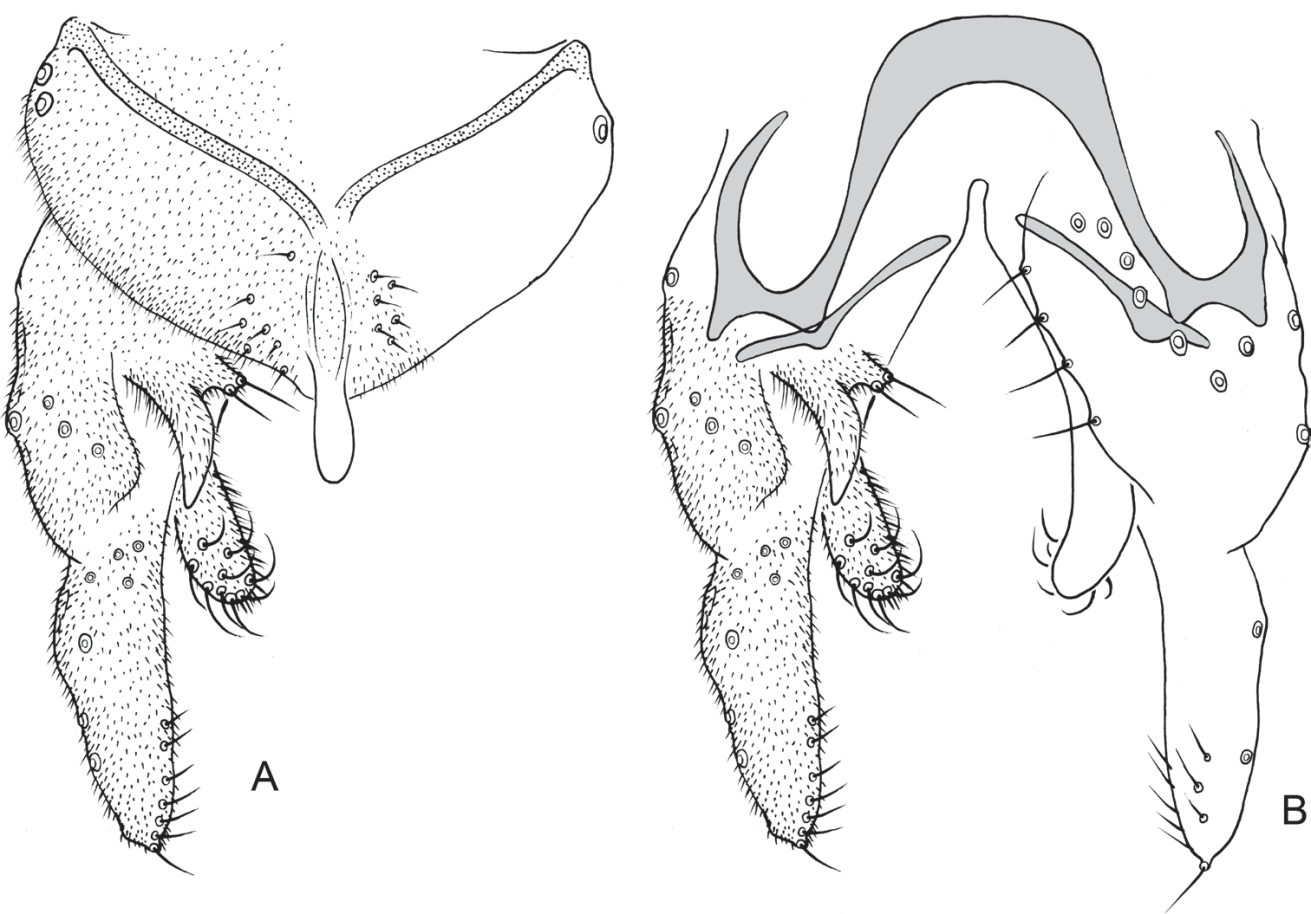
*Nilothauma duena* Roback, 1960, adult male **A** hypopygium, dorsal view **B** hypopygium with anal point and tergite IX removed, dorsal aspect to the left and ventral aspect to the right.

##### Re-description.

**Male imago (n = 2, unless otherwise stated).** Total length 2.37–2.76 mm. Wing length 1.56–1.68 mm. Total length/wing length 1.58–1.66. Wing length/length of profemur 2.60–2.62.

***Colouration*.** Head and thorax light brown; legs pale except for brown pigmentation in basal 1/3 and distal 1/5 of fore femur, basal 1/5 and distal 1/3 of fore tibia, distal 1/8 of mid- and hind femora, basal 1/8 and distal 1/8 of mid- and hind tibiae and entire tarsi. Abdominal tergites I–VIII with basal half light brown, distal half pale; hypopygium light brown. Wing membrane hyaline.

***Antenna*.**AR = 0.17–0.18. Thirteenth flagellomere 108–118 µm long.

***Head*.** Temporal setae 9–10 in single row. Clypeus with 13–15 setae. Tentorium 98 (1) µm long, maximum width 20 (1) µm. Stipes not measurable. Palp segment lengths (in µm): 17–25, 17–25, 32 (1), 35 (1), 62 (1). Third palpomere with 2 sensilla clavata subapically, longest 15 µm long. Fifth palpomere/third palpomere 1.92 (1).

***Thorax*.** Dorsocentrals 16–17 in single row, acrostichals 14–16, prealars 3. Scutellum with 2–3 setae.

***Wing*.**VR = 1.55–1.56. Brachiolum with 1 seta, R with 12–13 setae, R_1_ with 16–17, R_4+5_ with 5–22 setae, remaining veins bare.

***Legs*.** Spur of fore tibia 54–59 µm long including 15–20 µm long scale. Mid-tibia with 1 spur, 25–29 µm long; hind tibia with 2 spurs, 49–51 and 28–31 µm long. Combs of mid-tibia 15–20 µm long, of hind tibia 18–25 µm long. Width at apex of fore tibia 48–50 µm, of mid-tibia 48–50 µm, of hind tibia 49–59 µm. Lengths and proportions of legs as in Table 2.

**Table 2.** Lengths (in μm) and proportions of leg segments in *Nilothauma duena* Roback, 1960, adult males (n = 2).

	Fe	Ti	ta_1_	ta_2_	ta_3_	ta_4_
p_1_	601–640	453–502	660–690	374–376	276–278	207–217
p_2_	621–670	453–473	296–305	148–158	108–110	69–79
p_3_	739–778	670–699	404–443	217–236	207–210	148–150
	**ta_5_**	**LR**	**BV**	**SV**	**BR**	
p_1_	105–108	1.37–1.46	1.72–1.74	1.60–1.66	1.8–2.7	
p_2_	49–59	0.65–0.66	3.48–3.49	3.56–3.74	1.8–2.7	
p_3_	79–99	0.60–0.63	2.78–2.79	3.33–3.49	3.6–4.7	

***Hypopygium*** (Fig. 3A, B). Tergite IX without dorsal lobes, with triangular posterior margin with 13–15 weak setae along posterior margin to each side of base of anal point. Anal point spatulate, 22–30 µm long, maximum width 10–12 µm. Tergite bands well developed. Laterosternite IX with 1–2 setae. Phallapodeme 47–60 µm long; transverse sternapodeme 55–62 µm long. Gonocoxite 112–125 µm long. Inferior volsella straight, 40–45 µm long, 15–20 µm wide medially, with microtrichia and 10–11 strong, simple setae apically. Superior volsella tapering to apex, 20–37 µm long, 12–14 µm wide at base, covered with microtrichia and apparently bare at tip. Median volsella 7–10 µm long, with 3–4 setae, longest 20–22 µm long. Gonostylus 87–90 µm long, straight. HR = 1.29–1.43. HV = 2.72–3.17.

##### Female adult and immatures.

Unknown.

##### Remarks.

See remarks for *N. aleta* Roback, 1960.

##### Distribution

**(Fig. 16C).** The species was originally described by Roback (1960) from the Department of Huánuco, in the Peruvian Amazon; the range is now extended to Serra Bonita (Bahia State) and Serra do Mar (São Paulo State), in the Brazilian Atlantic Forest.

#### Nilothauma fittkaui

Taxon classificationAnimaliaDipteraChironomidae

(Soponis, 1987)

2786E149-32F5-521E-8564-A0D0F7549339

Figure 17A

##### Additional material.

2 males, slide-mounted: Brazil, São Paulo, Campos do Jordão, PE Campos do Jordão, Córrego Canhambora, 1538 m a.s.l., 22°41'44"S, 45°29'30"W, 13.i.2006, light trap, M.R. Spies leg. 1 male, slide-mounted, Costa Rica, La Selva, 03.iv.1993, Malaise trap, O.A. Sæther leg.

##### Distribution

**(Fig. 17A).** The species was described by Soponis (1987) from Amazonas and later recorded from Acre, Espírito Santo and Para States in Brazil and from Ecuador by Mendes and Andersen (2009). The range is now extended south to São Paulo State in Brazil and north to Costa Rica in Central America.

#### Nilothauma hamadae sp. nov.

Taxon classificationAnimaliaDipteraChironomidae

82206756-9AB8-5F2D-86C5-38ACF6C00463

http://zoobank.org/40ABE99F-793E-4688-9F2E-4D8B21B15E37

Figures 4A, B , 16A

##### Type material.

***Holotype*** male, slide-mounted: Brazil, Amazonas, Barcelos, Rio Aracá, Foz do Igarapé Cuieiras, 00°19'15"N, 63°16'15"W, 35 m a.s.l., 30.vii–01.viii.2009, light trap #11, N. Hamada et al. leg. (UFSC).

##### Etymology.

The specific epithet is a noun in the genitive case which honours Neusa Hamada for her great contribution to the knowledge of Amazonian Chironomidae.

**Figure 4.**
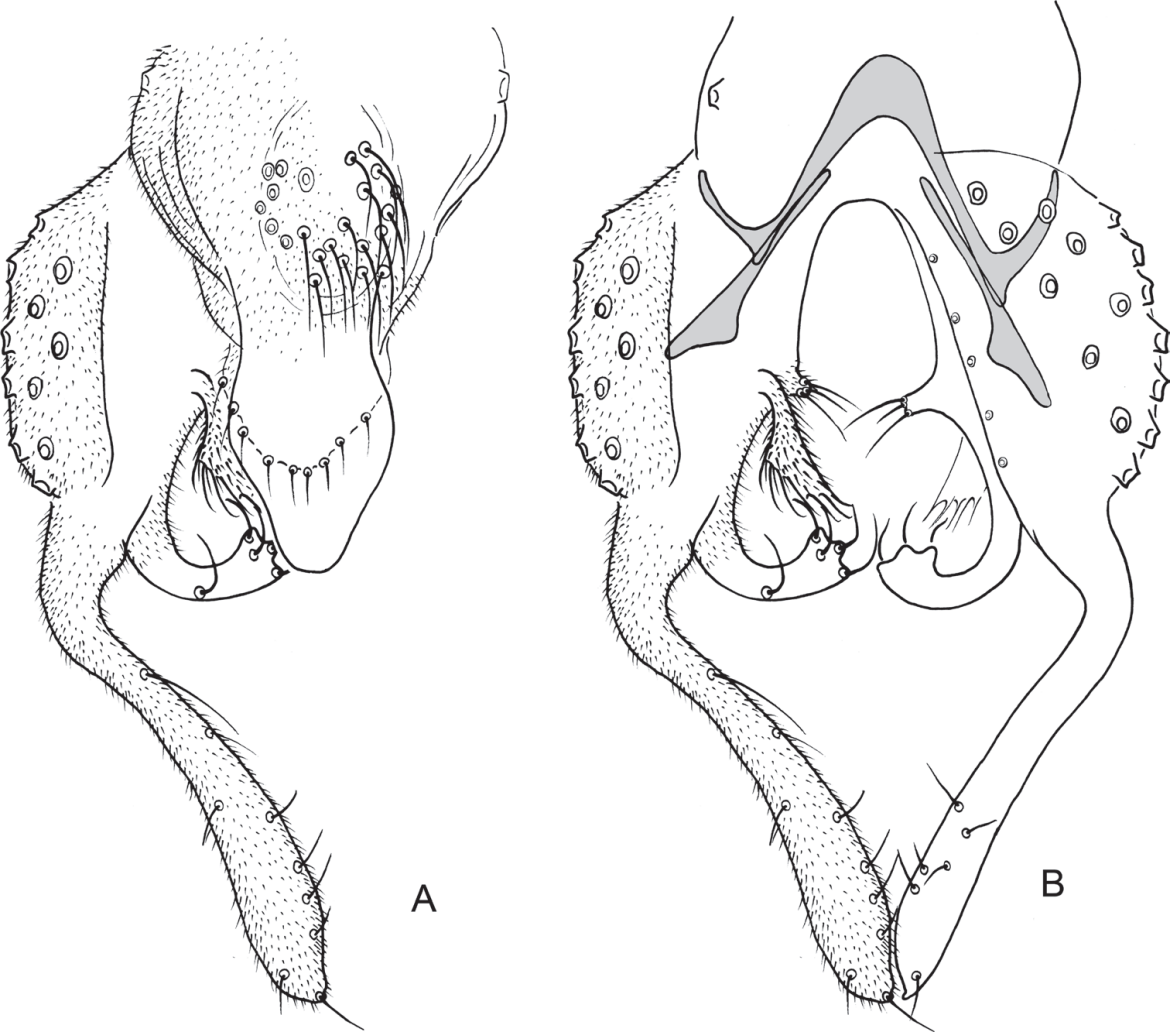
*Nilothauma hamadae* sp. nov. adult male **A** hypopygium, dorsal view **B** hypopygium with anal point and tergite IX removed, dorsal aspect to the left and ventral aspect to the right.

##### Diagnostic characters.

The male can be distinguished from its congeners by the combination of: tergite IX with single, median setose dorsal lobe, consisting of a low, but wide protuberance with about 25 strong setae; anal point very broad (about half the width of tergite IX) and lanceolate; median volsella separated from superior volsella.

##### Description.

**Male imago (n = 1).** Total length 1.94 mm. Wing length 1.13 mm. Total length/wing length 1.71. Wing length/length of profemur 2.30.

***Colouration*.** Head, thorax and abdomen medium brown. Legs light brown. Wing membrane hyaline.

***Antenna*.**AR = 0.40. Thirteenth flagellomere 217 µm long.

***Head*.** Temporal setae 7 in single row. Clypeus with 15 setae. Tentorium 75 µm long, maximum width 12 µm. Stipes 92 µm long. Palp segment lengths (in µm): 25, 25, 75, 127, 144. Third palpomere with 2 sensilla clavata subapically, longest 20 µm long. Fifth palpomere/third palpomere 1.93.

***Thorax*.** Dorsocentrals 7 in single row, acrostichals 10, prealars 2. Scutellum with 4 setae.

***Wing*.**VR = 1.37. Brachiolum with 1 seta, R with 13 setae, R_1_ with 7 setae, R_4+5_ with 14 setae, remaining veins bare.

***Legs*.** Spur of fore tibia 34 µm long including 12 µm long scale. Mid-tibia with 1 spur, 15 µm long; hind tibia with 2 spurs, 20 and 25 µm long. Combs of mid-tibia 12 µm long, of hind tibia 15 µm long. Width at apex of fore tibia 34 µm, of mid-tibia 34 µm, of hind tibia 39 µm. Lengths and proportions of legs as in Table 3.

**Table 3.** Lengths (in μm) and proportions of leg segments in *Nilothauma hamadae* sp. nov., adult male (n = 1).

	Fe	Ti	ta_1_	ta_2_	ta_3_	ta_4_
p_1_	493	364	–	–	–	–
p_2_	473	364	217	108	79	49
p_3_	532	542	315	167	158	99
	**ta_5_**	**LR**	**BV**	**SV**	**BR**	
p_1_	–	–	–	–	–	
p_2_	49	0.59	3.62	3.86	3.3	
p_3_	69	–	–	–	5.0	

***Hypopygium*** (Fig. 4A, B). Tergite IX narrow, tapering to apex, with central rounded lobe bearing 25 simple, strong setae; with 9 simple setae along posterior margin underneath the anal point. Anal point lanceolate, 42 µm long, 27 µm wide. Tergite bands lacking. Laterosternite IX with 1 seta. Phallapodeme 40 µm long; transverse sternapodeme 15 µm long. Gonocoxite 75 µm long. Inferior volsella strongly curved, 37 µm long, 7 µm wide medially, with microtrichia in basal half, with 4 simple setae apically and 1 simple seta subapically. Superior volsella 30 µm long, 4 µm wide at base, covered with microtrichia and fringed at apex. Median volsella 7 µm long, with 2 simple setae, longest 10 µm. Gonostylus 112 µm long, with basal half strongly curved and distal half straight. HR = 0.67. HV = 1.73.

##### Female adult and immatures.

Unknown.

##### Distribution

**(Fig. 16A).** Known from Barcelos (Amazonas State), in the Brazilian Amazon.

#### Nilothauma jaraguaense

Taxon classificationAnimaliaDipteraChironomidae

Mendes & Andersen, 2009

8B794255-4AF9-5557-BE38-5CFC1CA011E3

Figure 17D

##### Additional material.

1 male, slide-mounted: Brazil, São Paulo, Salesópolis, EB Boraceia, Rio Claro, Poço Verde, 18.ix.2002, light trap, A.S. Melo, C.G. Froehlich, R. Mariano, A. Prather & R. Blahnik leg. 1 male, slide-mounted: Brazil, São Paulo, Jundiaí, PE Serra do Japi, 23.ix.2008, light trap, R. Mariano & L.S. Lecci leg.

##### Distribution

**(Fig. 17D).** The species was described by Mendes and Andersen (2009), based on a single male from Parque Estadual do Jaraguá in São Paulo State, Brazil.

#### Nilothauma jupau sp. nov.

Taxon classificationAnimaliaDipteraChironomidae

95E0C167-6C7A-5002-8CDA-9F361FB77F77

http://zoobank.org/3F55ECB7-8CFC-4229-B33A-3FD9520A23B0

Figures 5A–C , 16A

##### Type material.

***Holotype*** male, slide-mounted: Brazil, Rondônia, Teixeirópolis, Balneário com Cachoeira, 10°55'20"S, 62°22'34"W, 03.ix.2012, light trap #13, N. Hamada, R. Boldrini, A.S. Fernandes & J.M. Cavalcante leg. (UFSC). ***Paratype***: 1 male, slide-mounted, same data as holotype (INPA).

##### Etymology.

The specific epithet honours the Jupaú, indigenous people from Rondônia State, Brazilian Amazon. The name is to be regarded as a noun in apposition.

##### Diagnostic characters.

The male can be distinguished from its congeners by the combination of: tergite IX with thorn and without setose dorsal lobe(s); anal point spatulate; wing with conspicuous dark markings; abdominal tergites II, III, VI, VII and VIII dark brown.

**Figure 5.**
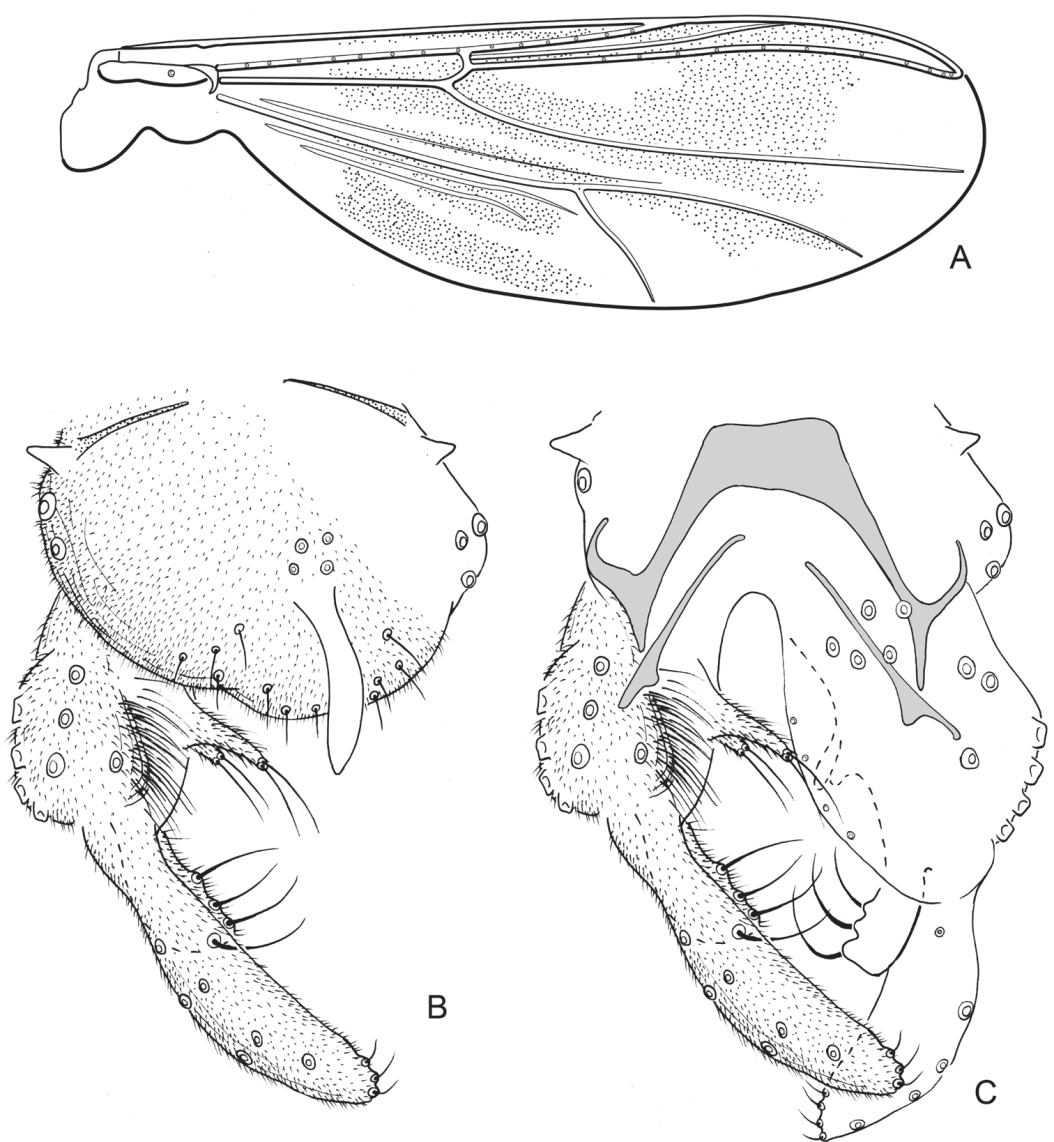
*Nilothauma jupau* sp. nov. adult male **A** wing **B** hypopygium, dorsal view **C** hypopygium with anal point and tergite IX removed, dorsal aspect to the left and ventral aspect to the right.

##### Description.

**Male imago (n = 2, unless otherwise stated).** Total length 2.04–2.14 mm. Wing length 1.07–1.14 mm. Total length/wing length 1.78–2.01. Wing length/length of profemur 2.03–2.27.

***Colouration*.** Head and thorax brown; legs pale, except for entire fore femur, distal half of mid- and hind femora and distal 1/3 of fore- and hind tibiae with brown pigmentation; abdomen pale, except for brown pigmentation in segments II, III, VI, VII and VIII. Wing membrane with extensive dark markings.

***Antenna*.**AR = 0.28–0.32. Thirteenth flagellomere 115–134 µm long.

***Head*.** Temporal setae 7–8 in single row. Clypeus with 20–24 setae. Tentorium 57–72 µm long, maximum width 15–17 µm. Stipes 95–100 µm long. Palp segment lengths (in µm): 13–14, 30–32, 85–95, 105–117, 115–134. Third palpomere with 2–3 sensilla clavata subapically, longest 14–15 µm. Fifth palpomere/third palpomere 1.21–1.69.

***Thorax*.** Dorsocentrals 6–7 in single row, acrostichals 8–14, prealars 2. Scutellum with 2 setae.

***Wing*** (Fig. 5A). VR = 1.63–1.64. Brachiolum with 1 seta, R with 8–10 setae, R_1_ with 6 setae, R_4+5_ with 11–15 setae, remaining veins bare.

***Legs*.** Spur of fore tibia 39–49 µm long including 20–25 µm long scale. Mid-tibia with 1 spur, 20–25 µm long; hind tibia with 2 spurs, 20–25 and 23–28 µm long. Combs of mid-tibia 15–20 µm long, of hind tibia 15–20 µm long. Width at apex of fore tibia 44 µm, of mid-tibia 39–44 µm, of hind tibia 47–51 µm. Lengths and proportions of legs as in Table 4.

**Table 4.** Lengths (in μm) and proportions of leg segments in *Nilothauma jupau* sp. nov., adult males (n = 2).

	Fe	Ti	ta_1_	ta_2_	ta_3_	ta_4_
p_1_	502–522	404–424	–	–	–	–
p_2_	483–522	335–345	180–187	87–89	57–59	37–39
p_3_	542–571	512–522	270–276	138–148	138–148	85–89
	**ta_5_**	**LR**	**BV**	**SV**	**BR**	
p_1_	–	–	–	–	–	
p_2_	28–30	0.54–0.56	3.22–3.31	4.22–4.56	1.8–2.8	
p_3_	59–69	0.53–0.54	2.93–3.23	3.82–3.96	1.5–3.2	

***Hypopygium*** (Fig. 5B, C). Tergite IX without dorsal lobes, with rounded posterior margin, with 4–5 clustered setae anteriorly to base of anal point and 8–12 weaker setae to each side of anal point. Anal point spatulate, 40–42 µm long, maximum width 7–10 µm. Tergite bands well developed. Laterosternite IX with 2–3 setae, with thorn. Phallapodeme 42–52 µm long; transverse sternapodeme 20–22 µm long. Gonocoxite 70–75 µm long, with longer microtrichia dorsomedially. Inferior volsella slightly curved, 30–32 µm long, 15–18 µm wide medially, with microtrichia and 5–6 simple setae in apical one third. Superior volsella slender, 12–20 µm long, 4–5 µm wide at base, covered with microtrichia and with 2 setae at apex, longest 8–13 µm. Median volsella consisting of small tubercle situated underneath superior volsella, 6–7 µm long, with 2 setae at apex, longest 6–7 µm long. Gonostylus 70–92 µm long, straight. HR = 0.81–1.00. HV = 2.21–3.05.

##### Female adult and immatures.

Unknown.

##### Distribution

**(Fig. 16A).** Known from Rondônia State, Brazilian Amazon.

#### Nilothauma karitiana sp. nov.

Taxon classificationAnimaliaDipteraChironomidae

837DF9A0-97D7-5106-89BE-73882F40A337

http://zoobank.org/20FE2D58-13AD-47D5-9414-9EB6B7BFC321

Figures 6A–C , 16C

##### Type material.

***Holotype*** male, slide-mounted: Brazil, Rondônia, Candeias do Jamari, Rio Preto, Ponte de Madeira, 08°52'40"S, 63°38'02"W, 19–20.vii.2012, light trap #01, R. Boldrini & A.S. Fernandes leg. (UFSC). ***Paratype***: 1 male adult, slide-mounted: BRAZIL, Amazonas, Barcelos, Rio Aracá, #9, 69 m a.s.l., 00°24'39"N, 63°23'12"W, 28.vii–06.viii.2009, light trap #3, N. Hamada et al. leg. (INPA).

##### Etymology.

The specific epithet honours the Karitiana, indigenous people from the Rio Jamari Basin in the Rondônia State (Brazil). The name is to be regarded as a noun in apposition.

**Figure 6.**
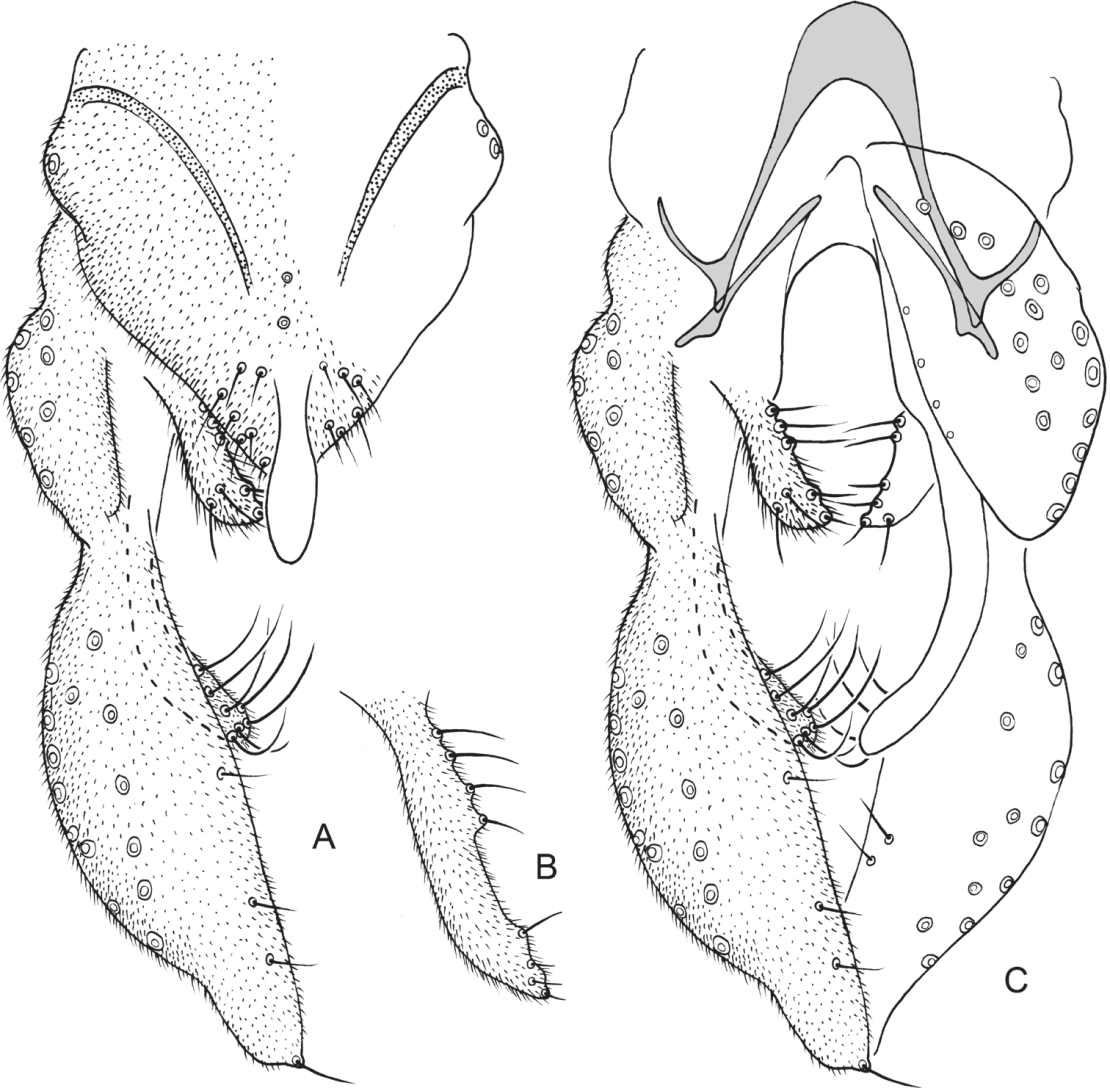
*Nilothauma karitiana* sp. nov. adult male **A** hypopygium, dorsal view **B** superior volsella, dorsal view **C** hypopygium with anal point and tergite IX removed, dorsal aspect to the left and ventral aspect to the right.

##### Diagnostic characters.

The male can be distinguished from its congeners by the combination of: tergite IX without setose dorsal lobe(s); anal point spatulate; wing unmarked; superior volsella fused to median volsella; gonostylus very long, narrow basally and apically, swollen medially.

##### Description.

**Male imago (n = 2, unless otherwise stated).** Total length 3.32–3.78 mm. Wing length 1.71–1.83 mm. Total length/wing length 1.94–2.07. Wing length/length of profemur 2.02–2.06.

***Colouration*.** Head, thorax, legs and abdomen uniformly light brown. Wing membrane hyaline.

***Antenna*.**AR = 0.22 (1). Thirteenth flagellomere 217 µm long.

***Head*.** Temporal setae 9 (1) in single row. Clypeus with 10–11 setae. Tentorium 105 (1) µm long, maximum width 17 (1) µm. Stipes 122 (1) µm long. Palp segment lengths (in µm): 25–35, 27–37, 60 (1), 80 (1), 154 (1). Third palpomere with 3 (1) sensilla clavata subapically, longest 20 (1) µm long. Fifth palpomere/third palpomere 2.57

***Thorax*.** Dorsocentrals 9–13 in single row, acrostichals 12, prealars 2–3. Scutellum with 2 setae.

***Wing*.**VR = 1.33–1.46. Brachiolum with 1 seta, R with 11–14 setae, R_1_ with 10–12 setae, R_4+5_ with 3–4 setae at apex, remaining veins bare.

***Legs*.** Spur of fore tibia 59–64 µm long including 17–20 µm long scale. Mid-tibia with 1 spur, 23–25 µm long; hind tibia with 2 spurs, 23–25 and 29–33 µm long. Combs of mid-tibia 15–20 µm long, of hind tibia 20–23 µm long. Width at apex of fore tibia 59 µm, of mid-tibia 59 µm, of hind tibia 64 µm. Lengths and proportions of legs as in Table 5.

**Table 5.** Lengths (in μm) and proportions of leg segments in *Nilothauma karitiana* sp. nov., adult males (n = 1–2).

	Fe	Ti	ta_1_	ta_2_	ta_3_	ta_4_
p_1_	847–887	729–808	1054	542	424	335
p_2_	739–798	532–561	374	158	108	69
p_3_	896–965	867–926	493–522	246–256	240–246	144–148
	**ta_5_**	**LR**	**BV**	**SV**	**BR**	
p_1_	138	1.30	1.91	1.61	2.2	
p_2_	49	0.67	4.51	3.63	3.2	
p_3_	95–99	0.56–0.57	3.01–3.27	3.58–3.62	4.0–5.0	

***Hypopygium*** (Fig. 6A, B). Tergite IX without dorsal lobes, tapering to apex, with 2–3 median and 8–9 setae to each side of anal point. Anal point spatulate, 55–57 µm long, 17–20 µm wide. Tergite bands well developed. Laterosternite IX with 1–2 setae. Phallapodeme 82–90 µm long; transverse sternapodeme 42–55 µm long. Gonocoxite 138–142 µm long. Inferior volsella slightly curved, 97–107 µm long, 17–20 µm wide medially, with microtrichia and 6–7 simple setae subapically. Superior volsella digitiform, 55–65 µm long, 17–20 µm wide at base, covered with microtrichia and with 4 setae apically. Median volsella fused to superior volsella, consisting of 2–4 small tubercles each bearing single, simple seta, longest 22–25 µm. Gonostylus 204–232 µm long, straight, narrow basally and apically, swollen medially. HR = 0.62–0.70. HV = 1.62–1.63.

##### Female adult and immatures.

Unknown.

##### Distribution

**(Fig. 16C).** Known from Rondônia and Amazonas States, in the Brazilian Amazon.

#### Nilothauma leccii sp. nov.

Taxon classificationAnimaliaDipteraChironomidae

6D99E53E-A830-5EC8-AF22-BF81F61B4241

http://zoobank.org/CBFC7D88-17AB-4BF8-9CBA-2995FDAB798B

Figures 7A, B , 16B

##### Type material.

***Holotype*** male, slide-mounted: Brazil, São Paulo, São Sebastião, Rio das Pedras, 23°44'27"S, 45°37'12"W, 28.x.2005, light trap, A.R. Calor et al. leg. (UFSC).

##### Etymology.

The specific epithet is a noun in the genitive case honouring Lucas Silveira Lecci, for his friendship and prolific fieldwork.

##### Diagnostic characters.

The male can be separated from its congeners by its large size combined with unmarked wing; spatulate anal point; superior volsella leaf-shapped; inferior volsella with strong, split setae and digitiform and strongly setose gonostylus.

**Figure 7.**
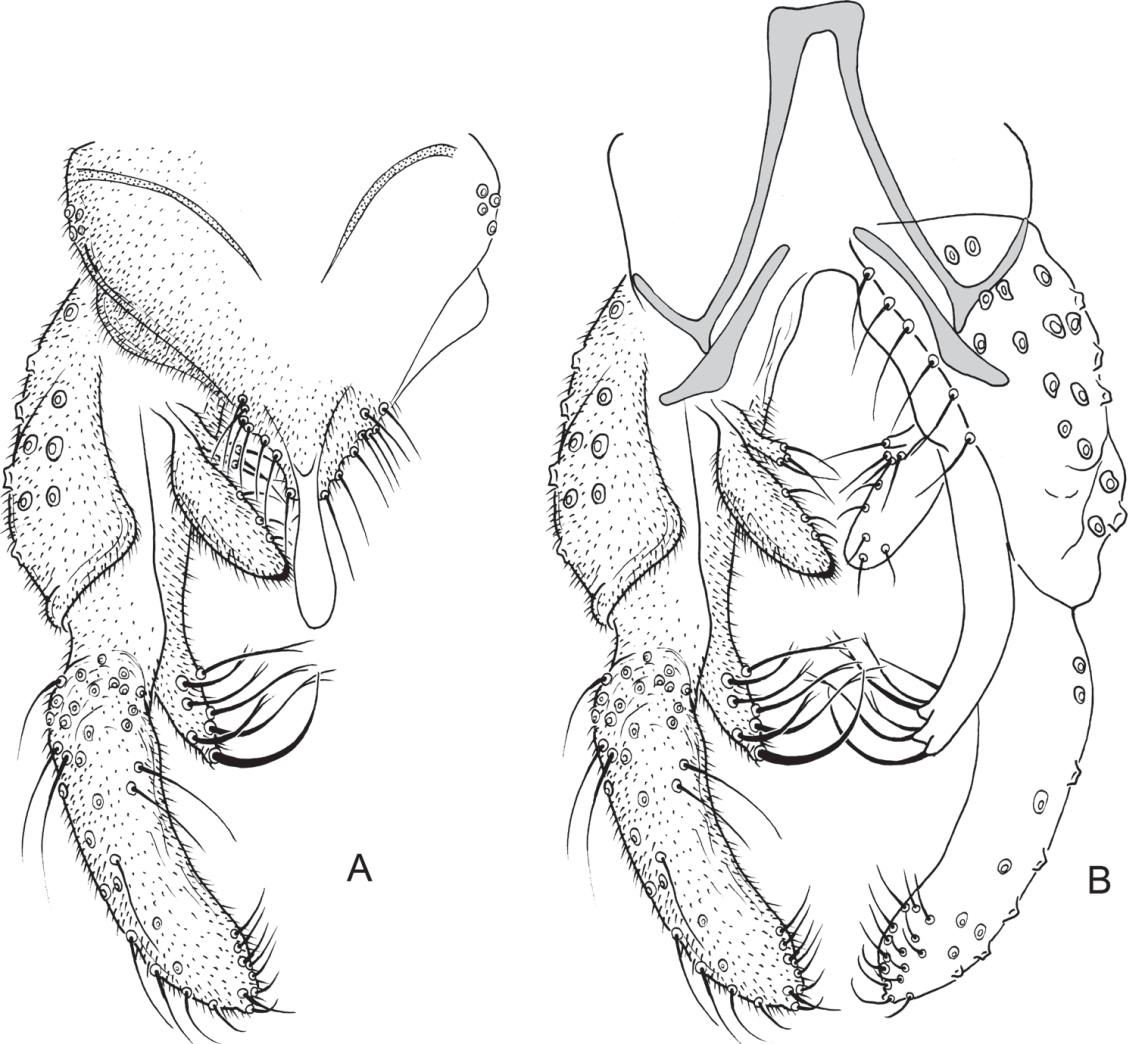
*Nilothauma leccii* sp. nov. adult male **A** hypopygium, dorsal view **B** hypopygium with anal point and tergite IX removed, dorsal aspect to the left and ventral aspect to the right.

##### Description.

**Male imago (n = 1).** Total length 4.35 mm. Wing length 1.96 mm. Total length/wing length 2.22. Wing length/length of profemur 2.45.

***Colouration*.** Thorax and legs brown, abdomen light brown. Wing membrane without dark markings.

***Antenna*.**AR = 0.16. Thirteenth flagellomere 124 µm long.

***Head*.** Temporal setae 6 in single row. Clypeus with 15 setae. Tentorium 113 µm long, maximum width 25 µm. Stipes not measurable. Palp segment lengths (in µm): 37, 33, 74, 107, 138. Third palpomere with 2 sensilla clavata subapically, longest about 25 µm long. Fifth palpomere/third palpomere 1.86.

***Thorax*.** Antepronotum with 4 setae. Dorsocentrals 17 partly biserial posterior, acrostichals 14, prealars 6. Scutellum with 13 setae.

***Wing*.**VR = 1.53. Brachiolum with 2 setae, R with 15 setae, R_1_ with 24 setae, R_4+5_ with 31 setae, remaining veins bare.

***Legs*.** Spur of fore tibia 65 µm long including 41 µm long scale. Mid-tibia with 1 spur, 47 µm long; hind tibia with 2 spurs, 43 and 65 µm long. Combs of mid-tibia 29 µm long, of hind tibia 47 µm long. Width at apex of fore tibia 69 µm, of mid-tibia 73 µm, of hind tibia 89 µm. Lengths and proportions of legs as in Table 6.

**Table 6.** Lengths (in μm) and proportions of leg segments in *Nilothauma leccii* sp. nov., adult male (n = 1).

	Fe	Ti	ta_1_	ta_2_	ta_3_	ta_4_
p_1_	801	670	972	490	384	286
p_2_	874	605	400	180	139	90
p_3_	964	989	596	302	261	155
	**ta_5_**	**LR**	**BV**	**SV**	**BR**	
p_1_	139	1.45	1.88	1.51	1.7	
p_2_	65	0.66	3.97	3.69	4.3	
p_3_	98	0.60	3.09	3.27	4.8	

***Hypopygium*** (Fig. 7A, B). Tergite IX without dorsal lobes, posterior margin subtriangular with 7 setae to each side of the anal point. Anal point spatulate, 59 µm long, 10 µm wide basally, 17 µm wide medially. Tergite bands not continuous. Laterosternite IX with 4 setae. Phallapodeme 61 µm long; transverse sternapodeme 35 µm long. Gonocoxite 171 µm long. Inferior volsella weakly curved, 113 µm long, 15 µm wide subapically, with microtrichia and 7 strong, apically split setae. Superior volsella leaf-shaped, 69 µm long, 10 µm wide at base, 23 µm wide medially, covered with microtrichia and with few weak setae ventrally and along inner margin. Median volsella narrow, 48 µm long, covered with microtrichia and with 4 setae on tubercles at apex, setae about 23 µm long. Gonostylus digitiform, strongly setose, 163 µm long, 37 µm wide medially. HR = 1.05. HV = 2.67.

##### Female adult and immatures.

Unknown.

##### Distribution

**(Fig. 16B).** Only known from São Paulo State in Brazil.

#### Nilothauma marianoi sp. nov.

Taxon classificationAnimaliaDipteraChironomidae

2EA33B93-E80C-5A75-BAC4-FF2F1DC0F297

http://zoobank.org/2F9585A3-AE43-47C7-BD91-C4EE9B23EEBC

Figures 8A, B , 16B

##### Type material.

***Holotype*** male, slide-mounted: Brazil, Bahia, Barreiras, Rio de Janeiro, cachoeira Acaba Vidas, 11°53'40"S, 45°36'57"W, 722 m a.s.l., 14.x.2008, light trap, A.R. Calor, R. Mariano & S. Mateus leg. (UFSC).

##### Etymology.

The specific epithet is a noun in the genitive case honouring Rodolfo Mariano, for his friendship and prolific fieldwork.

##### Diagnostic characters.

The male can be distinguished from its congeners by the combination of: wing without dark markings; tergite IX without setose dorsal lobe(s) or thorns, with single, strong median seta, with narrowly triangular posterior margin and small, apical, parallel-sided anal point.

**Figure 8.**
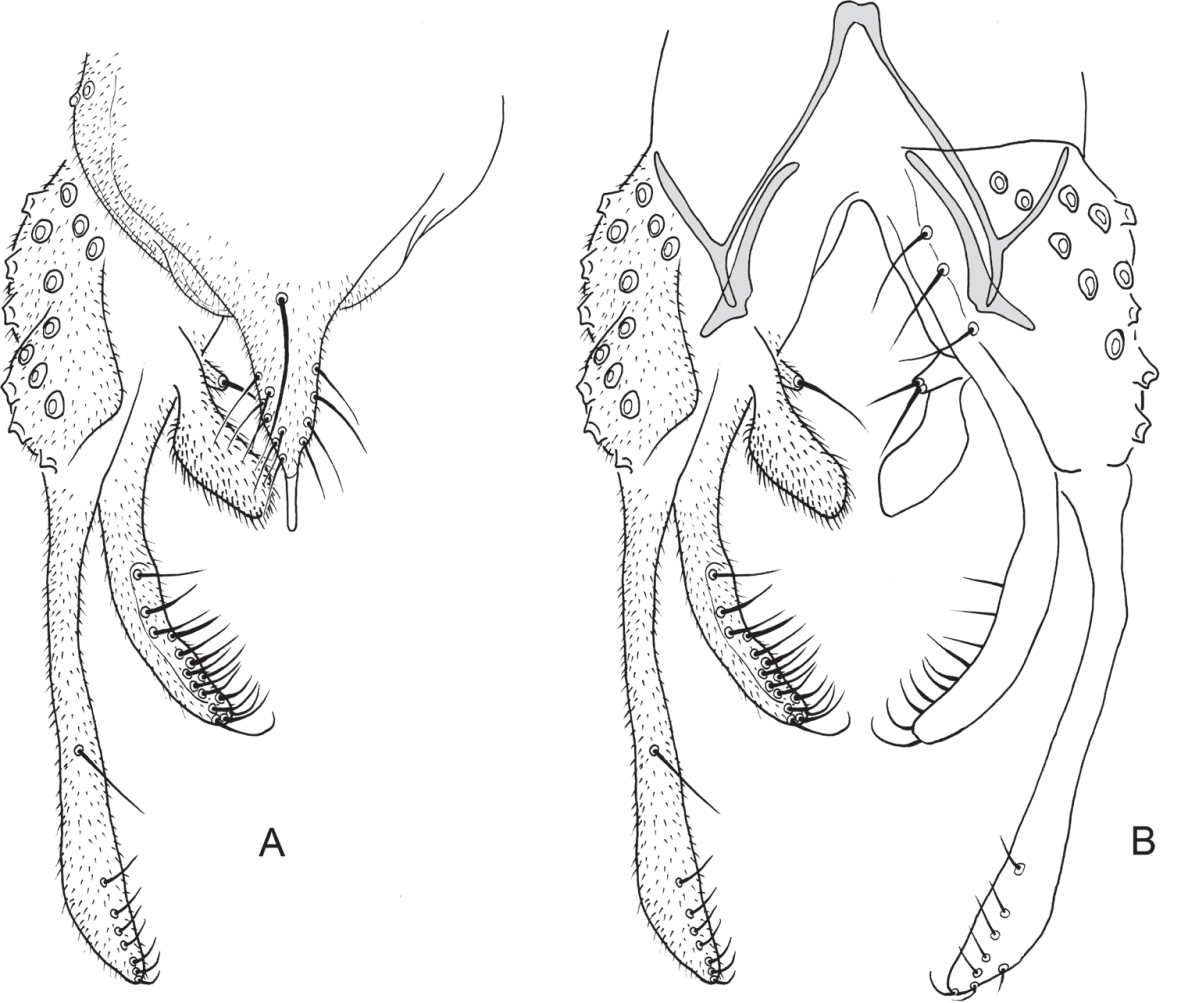
*Nilothauma marianoi* sp. nov. adult male **A** hypopygium, dorsal view **B** hypopygium with anal point and tergite IX removed, dorsal aspect to the left and ventral aspect to the right.

##### Description.

**Male imago (n = 1).** Total length 3.51 mm. Wing length 1.45 mm. Total length/wing length 2.43. Wing length/length of profemur 2.27.

***Colouration*.** Thorax and legs brown, abdomen light brown. Wing membrane without dark markings.

***Antenna*.**AR = 0.19. Thirteenth flagellomere 152 µm long.

***Head*.** Temporal setae 6 in partly double row. Clypeus with 9 setae. Tentorium 98 µm long, maximum width 18 µm. Stipes not measurable. Palp segment lengths (in µm): 17, 18, 44, 99, 117. Sensilla clavata on third palpomere not discernable. Fifth palpomere/third palpomere 2.66.

***Thorax*.** Dorsocentrals 6 in single row, acrostichals 4, prealars 2. Scutellum with 2 setae.

***Wing*.**VR = 1.39. Brachiolum with 1 seta, R with 8 setae, R_4+5_ with 1 apical seta, remaining veins bare.

***Legs*.** Spur of fore tibia 68 µm long including 28 µm long scale. Mid-tibia with 1 spur, 47 µm long; hind tibia with 2 spurs, 43 and 61 µm long. Combs of mid-tibia 21 µm long, of hind tibia 26 µm long. Width at apex of fore tibia 47 µm, of mid-tibia 48 µm, of hind tibia 52 µm. Lengths and proportions of legs as in Table 7.

**Table 7.** Lengths (in μm) and proportions of leg segments in *Nilothauma marianoi* sp. nov., adult male (n = 1).

	Fe	Ti	ta_1_	ta_2_	ta_3_	ta_4_
p_1_	637	458	645	401	310	221
p_2_	621	425	261	131	90	65
p_3_	694	686	384	180	180	114
	**ta_5_**	**LR**	**BV**	**SV**	**BR**	
p_1_	106	1.41	1.68	1.70	2.6	
p_2_	49	0.62	3.90	4.05	5.0	
p_3_	74	0.55	3.22	3.60	7.1	

***Hypopygium*** (Fig. 8A, B). Tergite IX without dorsal lobes, with single, median, strong setae, posterior margin narrowly subtriangular with 7 setae to each side. Anal point situated apically, small, parallel-sided with rounded apex, 14 µm long, 4 µm wide basally, 3 µm wide medially. Tergite bands lacking. Laterosternite IX with 2 setae. Phallapodeme 51 µm long; transverse sternapodeme 11 µm long. Gonocoxite 104 µm long. Inferior volsella weakly curved, 103 µm long, 11 µm wide subapically, with microtrichia and 18 setae in apical one-half. Superior volsella subquadrangular, 48 µm long, 17 µm wide medially, covered with microtrichia. Median volsella consisting of 14 µm long tubercle, covered with microtrichia and with 1 strong apical seta, setae about 19 µm long. Gonostylus nearly straight, 104 µm long, 10 µm wide medially, 17 µm wide subapically. HR = 0.72. HV = 2.44.

##### Female adult and immatures.

Unknown.

##### Distribution

**(Fig. 16B).** Only known from Bahia State in Brazil.

#### Nilothauma mateusi sp. nov.

Taxon classificationAnimaliaDipteraChironomidae

782674F3-D5A6-59FF-BE64-E4A6F7613293

http://zoobank.org/679EA037-9083-4F6D-9460-3F9F8E590EA2

Figures 9A, B ; 16B

##### Type material.

***Holotype*** male, slide-mounted: Brazil, Mato Grosso, Nova Xavantina, Fazenda Sr. Queté, Córrego Cachoeira, 14°32.817'S, 52°31.395'W, 16.x.2007, light trap, L.C. Pinho, S. Mateus, L. Torati & F.R. Silva leg. (UFSC).

##### Etymology.

The specific epithet is a noun in the genitive case honouring Sidnei Mateus, for his friendship and prolific fieldwork.

##### Diagnostic characters.

The male can be distinguished from its congeners by the combination of: wing without markings; tergite IX with pair of rounded lobes submedially with about 14 long setae; anal point parallel-sided; superior volsella small, subtriangular, projecting medially, with 2 setae on tubercles apically.

**Figure 9.**
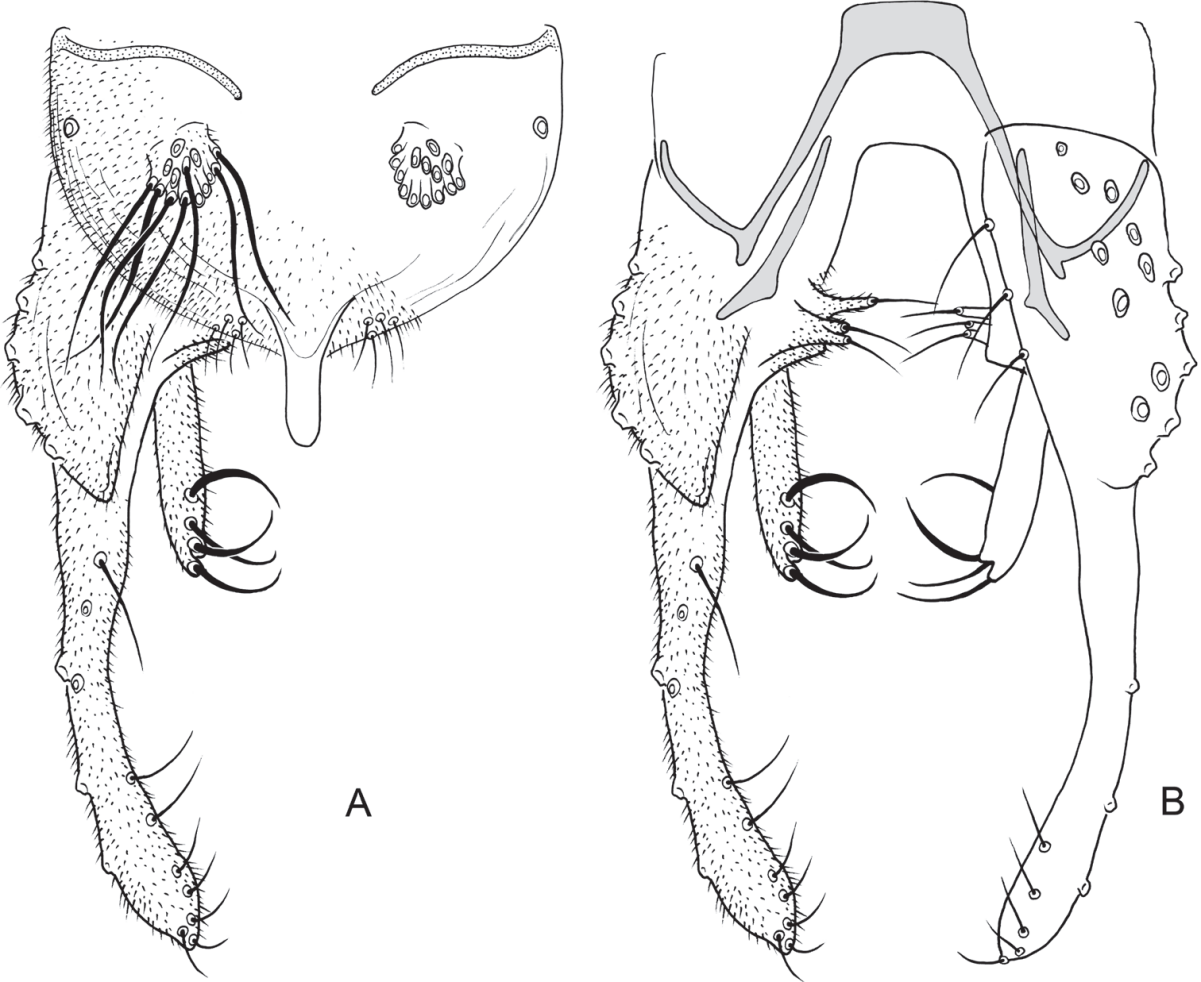
*Nilothauma mateusi* sp. nov. adult male **A** hypopygium, dorsal view **B** hypopygium with anal point and tergite IX removed, dorsal aspect to the left and ventral aspect to the right.

##### Description.

**Male imago (n = 1).** Total length 2.09 mm. Wing length 0.87 mm. Total length/wing length 2.40. Wing length/length of profemur 2.41.

***Colouration*.** Thorax and legs brown, abdomen light brown. Wing membrane without dark markings.

***Antenna*.**AR = 0.19. Thirteenth flagellomere 82 µm long.

***Head*.** Temporal setae 7 in single row. Clypeus with 9 setae. Tentorium 55 µm long, maximum width 12 µm. Stipes not measurable. Palp segment I–III lengths (in µm): 21, 19, 55; remaining palp segments lost. Third palpomere with 2 sensilla clavata subapically, longest about 10 µm.

***Thorax*.** Dorsocentrals 8 in single row, acrostichals 6, prealars 2. Scutellum with 2 setae.

***Wing*.**VR = 1.64. Brachiolum with 1 seta, R with 6 setae, R_1_ with 2 setae, R_4+5_ with 4 setae, remaining veins bare.

***Legs*.** Spur of fore tibia 37 µm long including 18 µm long scale. Mid-tibia with 1 spur, 23 µm long; hind tibia with 2 spurs, 19 and 28 µm long. Combs of mid-tibia 14 µm long, of hind tibia 17 µm long. Width at apex of fore tibia 29 µm, of mid-tibia 30 µm, of hind tibia 33 µm. Lengths and proportions of legs as in Table 8.

**Table 8.** Lengths (in μm) and proportions of leg segments in *Nilothauma mateusi* sp. nov., adult male (n = 1).

	Fe	Ti	ta_1_	ta_2_	ta_3_	ta_4_
p_1_	360	272	340	136	112	68
p_2_	352	248	140	64	52	32
p_3_	416	392	208	108	112	72
	**ta_5_**	**LR**	**BV**	**SV**	**BR**	
p_1_	40	1.25	2.73	1.86	2.0	
p_2_	24	0.56	4.30	4.29	2.8	
p_3_	40	0.53	3.06	3.88	4.6	

***Hypopygium*** (Fig. 9A, B). Tergite IX with 4 weak setae to each side of the anal point and pair of rounded lobes submedially, each with about 14 long setae, longest about 50 µm long. Anal point parallel-sided with rounded apex, 23 µm long, 12 µm wide basally, 8 µm wide medially. Tergite bands not continuous. Laterosternite IX with 1 seta. Phallapodeme 35 µm long; transverse sternapodeme 19 µm long. Gonocoxite 73 µm long. Inferior volsella straight, 43 µm long, 7 µm wide subapically, with microtrichia and 4 strong apical setae. Superior volsella projecting medially, subtriangular with two apical tubercles, 14 µm long, 7 µm wide at base, 4 µm wide subapically, covered with microtrichia and with 2 apical setae, longest 9 µm long. Median volsella consisting of single strong tubercle, about 12 µm long, with single 10 µm long setae at apex. Gonostylus curved, 101 µm long, 10 µm wide medially, 14 µm wide subapically. HR = 0.72. HV = 2.07.

##### Female adult and immatures.

Unknown.

##### Distribution

**(Fig. 16B).** Only known from Mato Grosso State in Brazil.

#### Nilothauma maya sp. nov.

Taxon classificationAnimaliaDipteraChironomidae

CD906BED-76E9-5CD5-9317-2534AA323D41

http://zoobank.org/0165F284-D9E4-4FF1-B207-C4EE505A0E00

**Figure 10.**
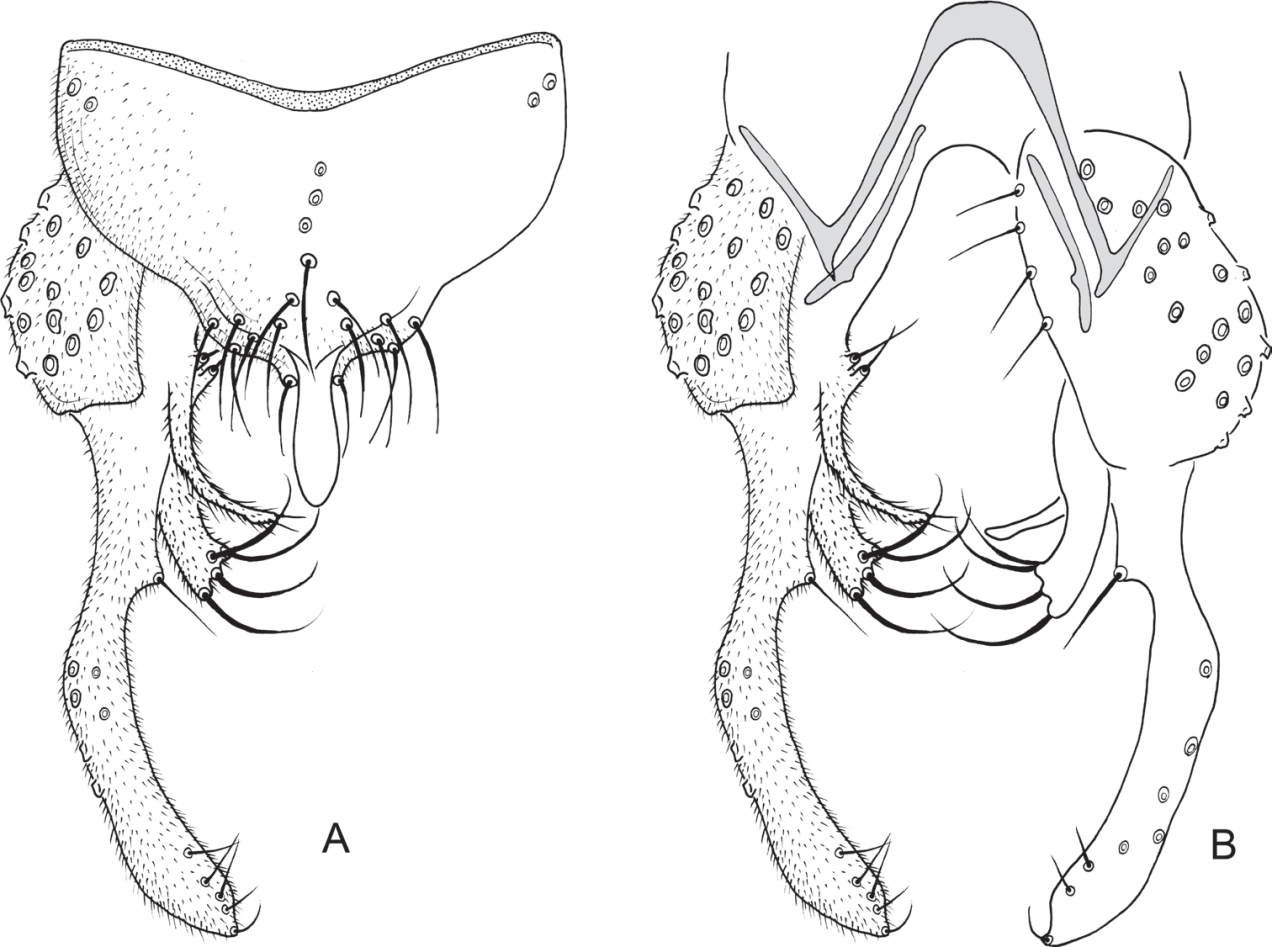
*Nilothauma maya* sp. nov. adult male **A** hypopygium, dorsal view **B** hypopygium with anal point and tergite IX removed, dorsal aspect to the left and ventral aspect to the right.

Figures 10A, B , 16B

##### Type material.

***Holotype*** male, slide-mounted: Mexico, Campeche, Calacmul, Ejido Nuevo Becan, El Chorro, 18°35'26"N, 89°15'29"W, 130 m a.s.l., 30.iv.1997, light trap, A. Contreras-Ramos et al. leg. (ZMBN).

##### Etymology.

Named after the Maya people, who used to live in the area. The name is to be regarded as a noun in apposition.

##### Diagnostic characters.

The male can be distinguished from its congeners by the combination of: pale brown species; wing without markings; tergite IX without setose dorsal lobe(s) or spine; anal point spatulate; superior volsella slender, curved, tapering; gonostylus curved, with strong setae on protruberance on inner margin in basal one-third.

##### Description.

**Male imago (n = 1).** Total length 2.43 mm. Wing length 1.12 mm. Total length/wing length 2.17. Wing length/length of profemur 2.15.

***Colouration*.** Pale brown. Wing membrane without dark markings.

***Antenna*.**AR = 0.18. Thirteenth flagellomere 112 µm long.

***Head*.** Temporal setae 4 in single row. Clypeus with 13 setae. Tentorium and stipes not measurable. Palp segment lengths (in µm): 21, 28, 65, 117, 144. Third palpomere with 2 sensilla clavata subapically, longest 19 µm long. Fifth palpomere/third palpomere 2.22.

***Thorax*.** Dorsocentrals 8 in single row, acrostichals 8, prealars 2. Scutellum with 2 setae.

***Wing*.**VR = 1.44. Brachiolum with 1 seta, R with 10 setae, R_1_ with 12 setae, R_4+5_ with 15 setae, remaining veins bare.

***Legs*.** Spur of fore tibia 48 µm long including 21 µm long scale. Mid-tibia with 1 spur, 26 µm long; hind tibia with 2 spurs, 25 and 33 µm long. Combs of mid-tibia 12 µm long, of hind tibia 17 µm long. Width at apex of fore tibia 36 µm, of mid-tibia 40 µm, of hind tibia 44 µm. Lengths and proportions of legs as in Table 9.

**Table 9.** Lengths (in μm) and proportions of leg segments in *Nilothauma maya* sp. nov., adult male (n = 1).

	Fe	Ti	ta_1_	ta_2_	ta_3_	ta_4_
p_1_	520	372	–	–	–	–
p_2_	484	308	208	76	56	32
p_3_	504	484	232	132	140	88
	**ta_5_**	**LR**	**BV**	**SV**	**BR**	
p_1_	–	–	–	–	–	
p_2_	28	0.68	5.21	3.81	3.4	
p_3_	48	0.48	2.99	4.26	4.7	

***Hypopygium*** (Fig. 10A, B). Tergite IX without lobes, with 8 setae above anal point and 5 somewhat weaker setae to each side of anal point. Anal point spatulate, 36 µm long, maximum width 11 µm. Tergite bands continuous. Laterosternite IX with 2 setae. Phallapodeme 48 µm long; transverse sternapodeme 27 µm long. Gonocoxite 104 µm long. Inferior volsella curved, 55 µm long, 14 µm wide subapically, with microtrichia and 4 strong apical setae. Superior volsella slender, curved, tapering, 54 µm long, 11 µm wide at base, 3 µm wide subapically, covered with microtrichia and with 2 weak apical setae. Median volsella consisting of two small tubercles, about 4 µm long, each with single setae at apex, longest 15 µm long. Gonostylus 131 µm long, curved, with single, strong setae on protuberance on inner margin at 27 µm from base, setae 21 mm long. HR = 0.79. HV = 1.85.

##### Female adult and immatures.

Unknown.

##### Remarks.

The slide is distorted and the drawings are composites of left and right side.

##### Distribution

**(Fig. 16B).** Only known from Campeche State in Mexico.

#### Nilothauma reissi

Taxon classificationAnimaliaDipteraChironomidae

(Soponis, 1987)

7BE2B9BF-55CC-507A-A138-5107F5779A94

Figure 17C

##### Additional material.

1 male, slide-mounted: Brazil, Santa Catarina, São Francisco do Sul, Distrito do Saí, 26°13'40"S, 48°40'50"W, CEPA Vila da Glória, 11–15.xi.2019, #143, light trap, L.C. Pinho et al. leg. 1 male, slide-mounted: Brazil, São Paulo, Santa de Rosa Viterbo, bridge at Tio Zito, 27.ix. 2000, light trap, H.F. Mendes & T. Andersen leg. 1 male, slide-mounted: Brazil, Mato Grosso, Ribeirão Cascalheira, Estrada Fazenda Manaus, 1° af. Rio Bonito, 12°57.088'S, 51°52.480'W, 08.x.2007, light trap, L.C. Pinho, S. Mateus, L. Torati & F.R. Silva leg.

##### Distribution

**(Fig. 17C).** The species was described from the Amazonas by Soponis (1987) and was later recorded from Minas Gerais and São Paulo States in northern and south-eastern Brazil by Mendes and Andersen (2009). The range is now extended to Mato Grosso and Santa Catarina States.

#### Nilothauma soka

Taxon classificationAnimaliaDipteraChironomidae

Andersen, Bello-González & Hagenlund, 2016

5D6079EA-F5B3-56B3-BC28-DE3C77DDEDD5

Figure 17C

##### Additional material.

2 males, slide-mounted: Brazil, Rondônia, Candeias do Jamari, Rio Preto, Ponte de Madeira, #01, 08°52'40"S, 63°38'02"W, 19–20.vii.2012, light trap, R. Boldrini & A.S. Fernandes leg. 2 males, slide-mounted: Brazil, Roraima, Boa Vista, Rio Cauamé, 02°52'06"N, 60°44'24"W, 9.iii.2009, light trap, L.M. Fusari leg. 2 males, slide-mounted: Brazil, Amazonas, Barcelos, Rio Aracá, #9, 69 m a.s.l., 00°24'39"N, 63°23'12"W, 28.vii–06.viii.2009, light trap #3, N. Hamada et al. leg. 3 males, slide-mouted: Brazil, Amazonas, Barcelos, Rio Aracá, Foz do Igarapé Cuieiras, 00°19'15"N, 63°16'15"W, 35 m a.s.l., 30.vii–01.viii.2009, light trap #11, N. Hamada et al. leg.

##### Distribution

**(Fig. 17C).** The species was originally described from the Amazonas State by Andersen et al. (2016); the range is now extended to the Rondônia and Roraima States in the Brazilian Amazon.

#### Nilothauma strebulosum

Taxon classificationAnimaliaDipteraChironomidae

(Adam & Sæther, 2000)

951C762D-5190-50DB-ABE8-555FF7137437

Figure 16D

##### Additional material.

1 male, slide-mounted: Brazil, Mato Grosso, Nova Xavantina, Fazenda Sr. Queté, Córrego Voadeira, 14°32.187'S, 52°30.902'W, 16.x.2007, light trap, L.C. Pinho, S. Mateus, L. Torati & F.R. Silva leg.

##### Distribution

**(Fig. 16D).** The species was originally described from Costa Rica by Adam and Sæther (2000); the range is now extended to Mato Grosso State, central Brazil.

#### Nilothauma terena sp. nov.

Taxon classificationAnimaliaDipteraChironomidae

622463DF-AC49-5E3A-AD57-3B1BC84A1198

http://zoobank.org/140D5884-DBE1-403A-AD55-6AD631840573

**Figure 11.**
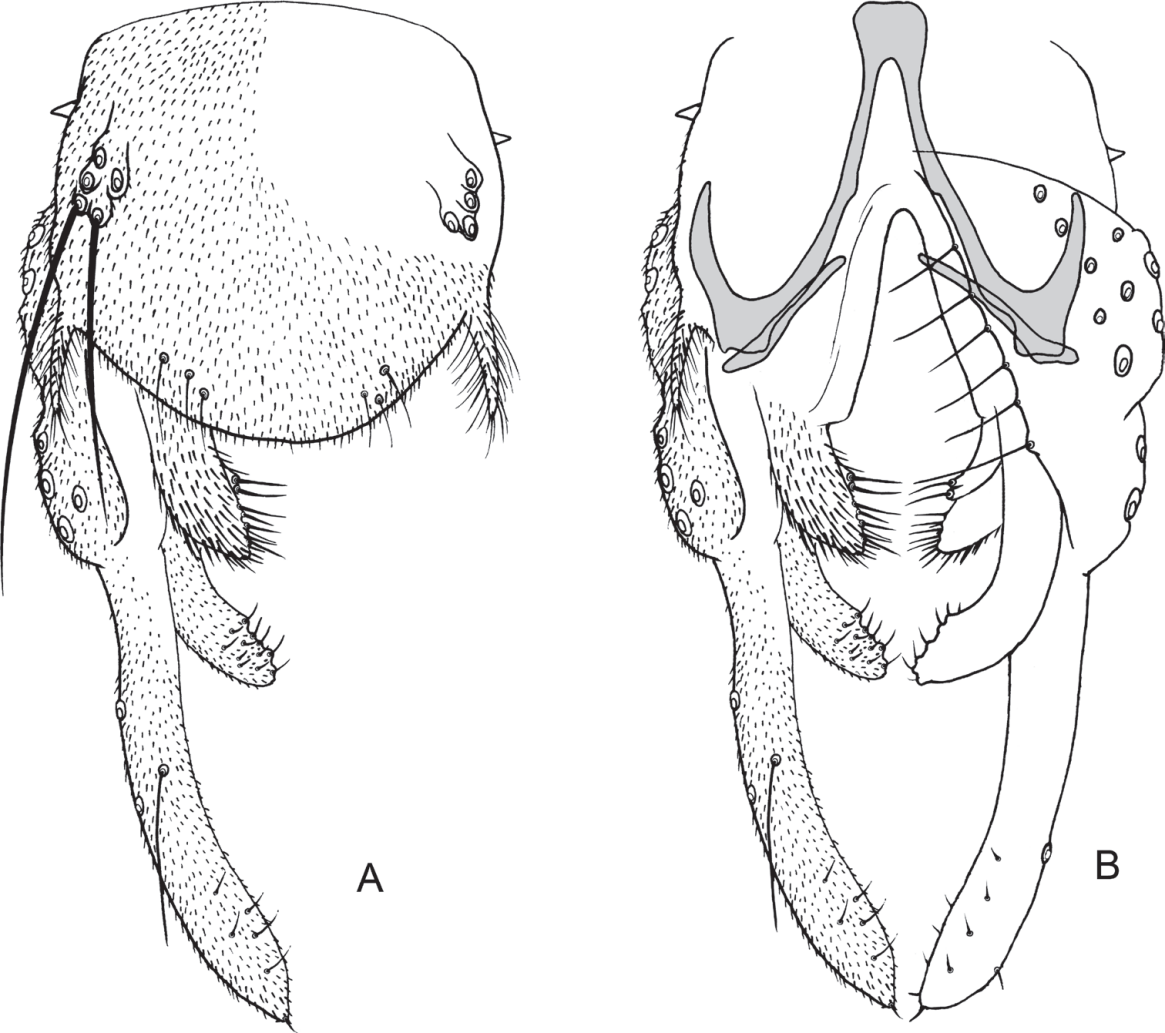
*Nilothauma terena* sp. nov. adult male **A** hypopygium, dorsal view **B** hypopygium with d tergite IX removed, dorsal aspect to the left and ventral aspect to the right.

**Figure 12.**
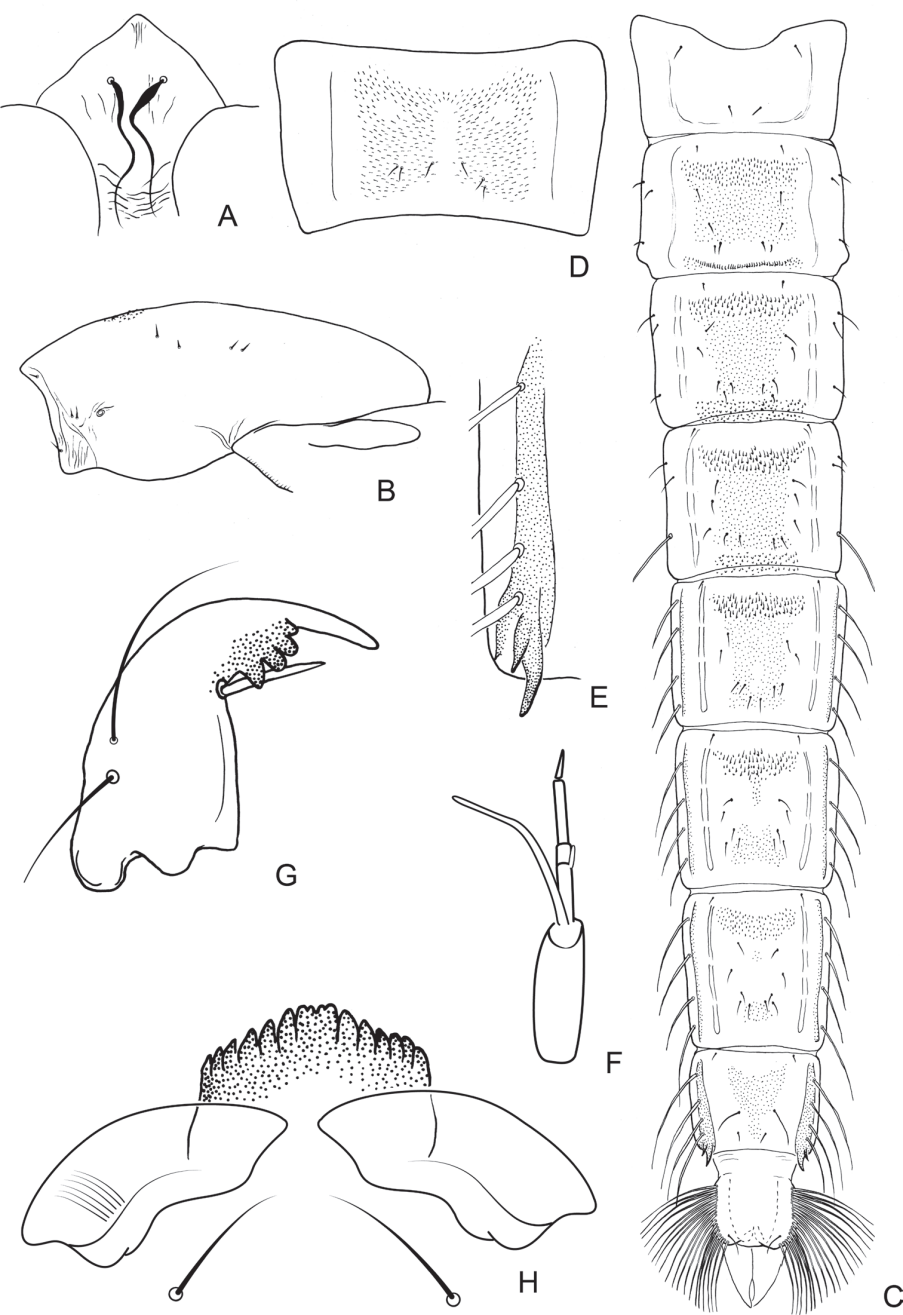
*Nilothauma terena* sp. nov. pupa (**A–E**) and larva (**F–H**) **A** frontal apotome **B** thorax **C** abdomen, dorsal view **D** sternite I **E** paratergite VIII **F** antenna **G** mandible **H** mentum and ventromental plates.

Figures 11A, B , 12A–H , 16A

##### Type material.

***Holotype*** male with larval and pupal exuvia, slide-mounted: Brazil, São Paulo, São Carlos, Campus UFSCar, Córrego do Fazzari, 21°59'S, 47°54'W, 11.ix.2008, L.C. Pinho & F.L. Silva leg. (UFSC).

##### Etymology.

The specific epithet honours the Terena indigenous people from São Paulo State (Brazil). The name is to be regarded as a noun in apposition.

##### Diagnostic characters.

The male can be distinguished from its congeners by having tergite IX with broadly rounded posterior margin without anal point, with anterolateral thorns, with dorsolateral lobes with few, strong setae and posteriolateral, narrowly subtriangular projection. The pupa can be recognised by having long, taeniate frontal setae and sternite I with extensive shagreen. The larva can be recognised by apparently having antenna with five segments only and by having mentum and inner teeth of mandible somewhat darker pigmented.

##### Description.

**Male imago (n = 1).** Total length 2.67 mm. Wing length 1.21 mm. Total length/wing length 2.21. Wing length/length of profemur 2.11.

***Colouration*.** Thorax and legs brown, abdomen light brown. Wing membrane without dark markings.

***Antenna*.**AR = 0.21. Thirteenth flagellomere 120 µm long.

***Head*.** Temporal setae 5 in single row. Clypeus with 13 setae. Tentorium 62 µm long, maximum width 15 µm. Stipes not measurable. Palp segment lengths (in µm): 20, 23, 57, 92, 106. Third palpomere with 2 sensilla clavata subapically, longest about 25 µm. Fifth palpomere/third palpomere 1.87.

***Thorax*.** Dorsocentrals 5 in single row, acrostichals 4, prealars 2. Scutellum with 2 setae.

***Wing*.**VR = 1.46. Brachiolum with 1 seta, R with 6 setae, R_1_ with 1 seta, R_4+5_ with 1 apical seta, remaining veins bare.

***Legs*.** Spur of fore tibia 62 µm long including 32 µm long scale. Mid-leg missing; hind tibia with 2 spurs, 28 and 46 µm long. Combs of hind tibia 18 µm long. Width at apex of fore tibia 37 µm, of hind tibia 39 µm. Lengths and proportions of legs as in Table 10.

**Table 10.** Lengths (in μm) and proportions of leg segments in *Nilothauma terena* sp. nov., adult male (n = 1).

	**Fe**	**Ti**	**ta_1_**	**ta_2_**	**ta_3_**	**ta_4_**
p_1_	572	359	–	–	–	–
p_2_	–	–	–	–	–	–
p_3_	563	574	310	155	147	90
	**ta_5_**	**LR**	**BV**	**SV**	**BR**	
p_1_	–	–	–	–	–	
p_2_	–	–	–	–	–	
p_3_	49	0.57	3.22	3.58	7.1	

***Hypopygium*** (Fig. 11A, B). Tergite IX with rounded posterior margin with altogether 6 marginal setae in two posteriolateral groups; with dorsolateral lobes with 5 strong setae, longest setae 65 µm long; with posteriolateral, narrowly subtriangular projections, 14 µm long, 6 µm wide at base. Tergite band not apparent. Laterosternite IX without setae; with small anteriolateral thorn. Phallapodeme 33 µm long; transverse sternapodeme 11 µm long. Gonocoxite 83 µm long. Inferior volsella digitiform, curved, 55 µm long, 11 µm wide medially, with microtrichia and 12 short setae subapically. Superior volsella 29 µm long, 12 µm wide at base, 13 µm wide medially, covered with microtrichia and with marginal setae. Median volsella subtriangular with 2 apical setae on small tubercles, setae about 6 µm long. Gonostylus weakly curved, 97 µm long, 14 µm wide medially. HR = 0.85. HV = 2.75.

##### Female adult.

Unknown.

##### Pupa

**(n = 1).** Total length 3.59 mm. Exuviae pale brown.

***Cephalothorax*** (Fig. 12A, B). Frontal apotome (Fig. 12A) with few wrinkles, frontal setae taeniate, 154 μm long. Thoracic horn not discernible; basal ring oval, 13 μm in diameter. Scutum with field of few weak tubercles. Antepronotals 2; precorneals 2; dorsocentrals 4, Dc1 39 μm in front of Dc2, Dc2 96 μm in front of Dc3, Dc3 23 μm in front of Dc4.

***Abdomen*** (Fig. 12C–E). Tergite I bare; tergites II–VI with transverse anterior band of somewhat stronger spinules, merging with median field of finer shagreen; anterior band of shagreen on tergite VI separated from posterior shagreen patch; tergite VII with anterior and posterior shagreen patches; tergite VIII with anterior shagreen patch connected with narrow posterior field of finer shagreen; tergite IX bare. Sternite I (Fig. 12D) with extensive shagreen; sternite II–VII bare; sternite VIII with narrow, longitudinal field of fine shagreen. Tergite II with 159 μm long row of 36 hooks, each hook about 8 μm long. Conjunctives III/IV and IV/V with spinules extending on to preceding segment. Pedes spurii B weakly developed on segment II. Anal comb 51 μm long, consisting of 3 spurs.

***Abdominal setation*.** Lateral setae on segments I–VIII as: 0, 3, 3, 3, 4, 4, 4, 4; posterior lateral seta on tergite IV and all lateral setae on tergites V–VIII taeniate, remaining setae hair-like. All tergites with 1 pair of O setae.

***Anal lobe*.** As long as broad, with 1 taeniate dorsal setae and complete fringe of 19 taeniae on each side, longest 170 μm. Male genital sac over-reaches anal lobe by 119 μm.

##### Fourth instar larva

**(n = 1)**. Head capsule 228 μm long. Postmentum 145 μm long.

***Head*.** Antenna (Fig. 12F) apparently with five segments only, length of antennal segments (in μm): 19, 10, 4, 11, 6. AR = 0.61. Basal antennal segment 10 μm wide; blade 32 μm long; accessory blade about 5 μm long. Premandible not measurable, teeth not discernible. Mandible (Fig. 12G) 69 μm long, seta subdentalis 19 μm long, inner teeth somewhat darker pigmented. Mentum (Fig. 12H) somewhat darker pigmented, 41 μm wide; middle part 10 μm wide with 2 minute inner teeth and pair of slightly larger lateral teeth; with 6 pairs of pointed, medially curved lateral teeth. Ventromental plates 98 μm wide, medially separated by 10 μm. Seta submenti 46 μm long.

***Abdomen*.** Lost.

##### Distribution

**(Fig. 16A).** Known from São Paulo State, south-eastern Brazil.

#### Nilothauma txukuyana sp. nov.

Taxon classificationAnimaliaDipteraChironomidae

2003CFA5-D8EC-578C-92A9-7D7FE307B04B

http://zoobank.org/8D8DBE02-E551-48F2-AFD8-DFF0B4263FCE

**Figure 13.**
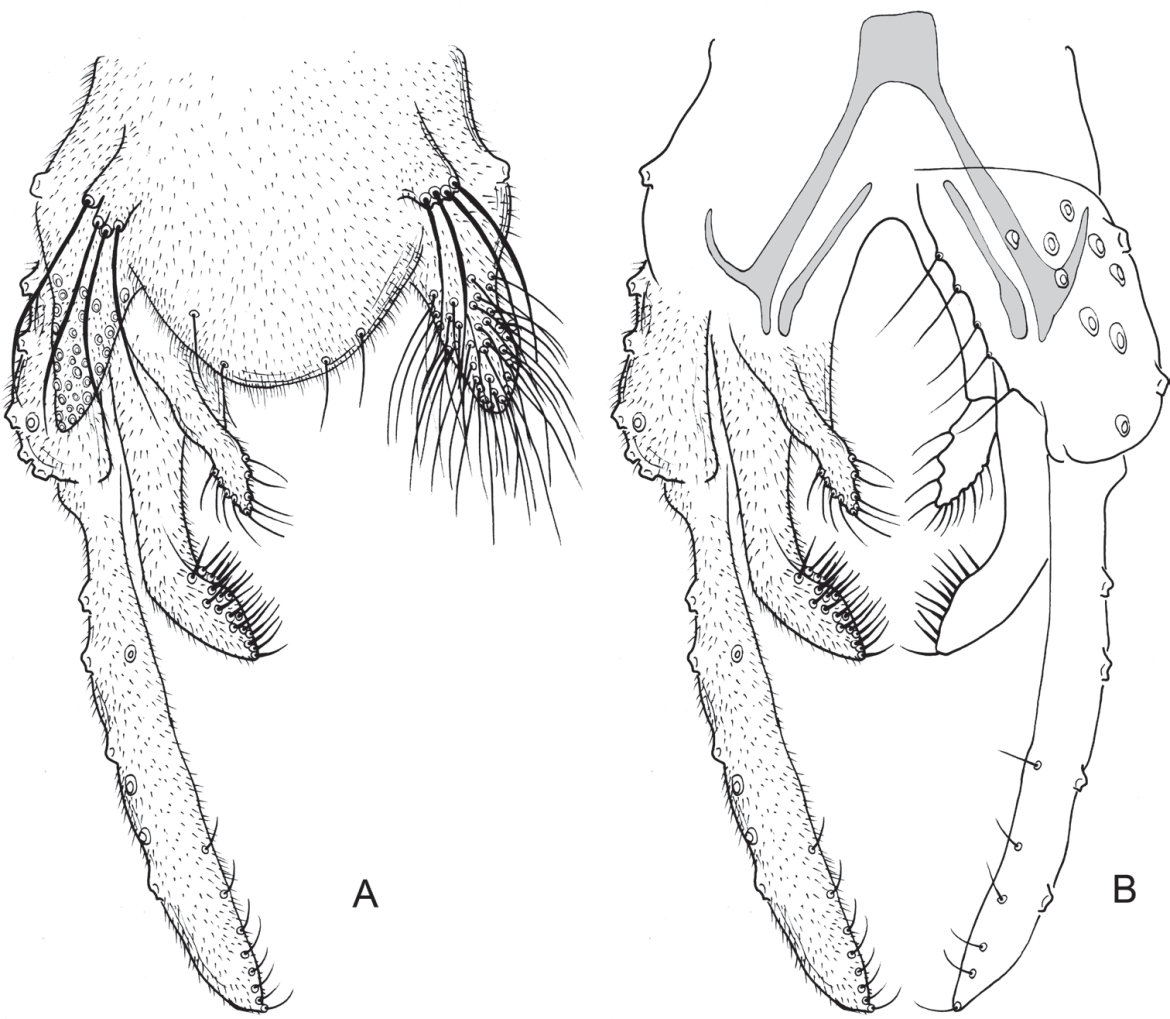
*Nilothauma txukuyana* sp. nov. adult male **A** hypopygium, dorsal view **B** hypopygium with tergite IX removed, dorsal aspect to the left and ventral aspect to the right.

Figures 13A, B , 16B

##### Type material.

***Holotype*** male, slide-mounted: Brazil, Pará, Rio Paru do Oeste, Malloca Apicó, 20.iv.1962, at light, E.J. Fittkau leg. (A 366-1, ZSM). ***Paratypes***: 15 males, same data as holotype (ZSM, ZMBN, UFSC).

##### Etymology.

The specific epithet honours the Txukuyana, indigenous people from Amazonas and Pará States in Brazil and from Suriname. The name is to be regarded as a noun in apposition.

##### Diagnostic characters.

The male can be distinguished from its congeners by having tergite IX with broadly-rounded posterior margin without anal point, with dorsolateral lobes with few, strong setae and posteriolateral, strongly setose, subtriangular projection.

##### Description.

**Male imago (n = 5–8).** Total length 2.17–2.44, 2.25 mm. Wing length 1.00–1.09, 1.05 mm. Total length/wing length 2.02–2.33, 2.16. Wing length/length of profemur 2.16–2.26, 2.21.

***Colouration*.** Head, thorax and legs brown; abdomen light brown. Wing membrane without dark markings.

***Antenna*.**AR = 0.26–0.29, 0.28. Thirteenth flagellomere 132–156, 145 µm long.

***Head*.** Temporal setae 6–10, 7 in single row. Clypeus with 13–16, 15 setae. Tentorium 69–83, 77 µm long, maximum width 14–19, 19 µm. Stipes not measurable. Palp segment lengths (in µm): 18–25, 21; 23–28, 26; 56–60, 57; 76–81, 79; 102–115, 107. Third palpomere with 2 sensilla clavata subapically, longest about 15 µm long. Fifth palpomere/third palpomere 1.72–1.98, 1.85.

***Thorax*.** Dorsocentrals 4–7, 6 in single row, acrostichals apparently 4–6, 5 anterior, prealars 1–2, 2. Scutellum with 2 setae.

***Wing*.**VR = 1.44–1.54, 1.50. Brachiolum with 1 seta, R with 7–9, 8 setae, R_1_ with 5–8, 7 setae, R_4+5_ with 9–13, 11 setae apically, remaining veins bare.

***Legs*.** Spur of fore tibia 48–54, 52 µm long including 20–25, 22 µm long scale. Mid-tibia with 1 spur, 29–35, 32 µm long; hind tibia with 2 spurs, 22–28, 25 and 33–39, 36 µm long. Combs of mid-tibia 17–18, 18 µm long, of hind tibia 19–22, 21 µm long. Width at apex of fore tibia 33–37, 35 µm, of mid-tibia 33–38, 36 µm, of hind tibia 39–41, 40 µm. Lengths and proportions of legs as in Table 11.

**Table 11.** Length (in µm) and proportions of legs of *Nilothauma txukuyana* sp. nov., adult males (n = 5–7).

	Fe		Ti		ta_1_	ta_2_
p_1_	457–523, 482		319–359, 338		474–547, 515	194–221, 203
p_2_	449–474, 462		286–310, 301		147–179, 162	65–74, 69
p_3_	507–556, 529		458–482, 467		245–278, 263	114–139, 127
	**ta_3_**		**ta_4_**		**ta_5_**	**LR**
p_1_	147–163, 157		106–123, 114		57–65, 59	1.49–1.59, 1.52
p_2_	41–49, 46		25–33, 29		24–32, 26	0.50–0.58, 0.54
p_3_	114–147, 129		73–90, 78		41–49, 47	0.54–0.58, 0.56
		**BV**		**SV**		**BR**
p_1_		2.47–2.54, 2.51		1.54–1.64, 1.59		2.43–3.46, 2.71
p_2_		5.13–5.73, 5.45		4.36–5.06, 4.73		2.77–3.76, 3.38
p_3_		3.20–3.57, 3.30		3.70–4.00, 3.79		4.33–5.00, 4.60

***Hypopygium*** (Fig. 13A, B). Tergite IX with rounded posterior margin with 4–7, 5 marginal setae; with dorsolateral lobes with 3–4, 4 strong setae, longest setae 44–55, 50 µm long; with posteriolateral subtriangular, strongly setose projection, 35–41, 38 µm long, 22–25, 24 µm wide at base. Tergite band not apparent. Laterosternite IX with single setae. Phallapodeme 47–55, 49 µm long; transverse sternapodeme 17–19, 18 µm long. Gonocoxite 83–89, 87 µm long. Inferior volsella digitiform, curved, 62–72, 67 µm long, 11–14, 12 µm wide medially, with microtrichia and 13–17, 15 setae subapically. Superior volsella 25–29, 27 µm long, 8–11, 10 µm wide at base, 7–10, 9 µm wide medially, covered with microtrichia and with marginal setae. Median volsella small, broadly triangular, apparently without setae. Gonostylus nearly straight, 126–135, 130 µm long, 17–19, 18 µm wide medially. HR = 0.65–0.68, 0.66. HV = 1.78–1.87, 1.82.

##### Female imago and immatures.

Unknown.

##### Distribution

**(Fig. 16B).** Known from Pará State, Brazil.

#### Nilothauma werekena sp. nov.

Taxon classificationAnimaliaDipteraChironomidae

CED00D36-A5D8-5E7F-9F07-F32D60890CA9

http://zoobank.org/2B7C1444-B834-4FAD-BE69-E0417DD42812

Figures 14A–C , 16D

##### Type material.

***Holotype*** male, slide-mounted: Brazil, Amazonas, Barcelos, Rio Aracá, Foz do Igarapé Cuieiras, #11, 00°19'15"N, 63°16'15"W, 35 m a.s.l., 30.vii–01.viii.2009, light trap, N. Hamada et al. leg. (UFSC). ***Paratypes***: 4 males, slide-mounted, same data as holotype (INPA). 2 males, slide-mounted, same data as previous, except: #9, 00°24'39"N, 63°23'12"W, 69 m a.s.l., 28.vii–06.viii.2009, light trap #3, N. Hamada et al. leg (MZSP).

**Figure 14.**
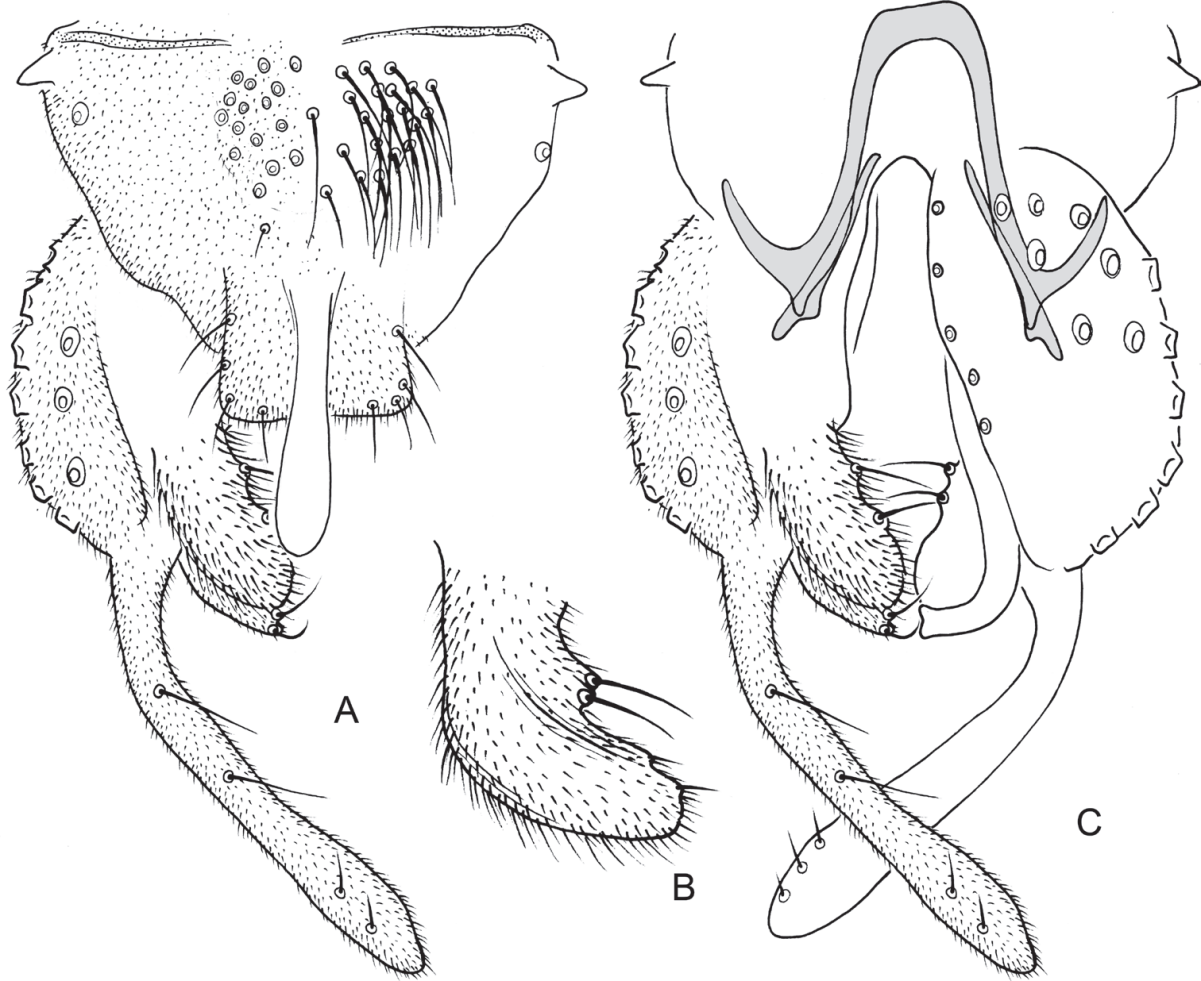
*Nilothauma werekena* sp. nov. adult male **A** hypopygium, dorsal view **B** superior volsella, dorsal view **C** hypopygium with anal point and tergite IX removed, dorsal aspect to the left and ventral aspect to the right.

##### Etymology.

The specific epithet honours the Werekena indigenous people from the Rio Negro Basin in the Amazon. The name is to be regarded as a noun in apposition.

##### Diagnostic characters.

The male can be distinguished from its congeners by the combination of: tergite IX with one low, but wide median dorsal protruberance with about 30 strong setae; anal point spatulate; superior volsella covered with microtrichia, fused to median volsella; laterosternite IX with thorn.

##### Description.

**Male imago (n = 5–7, unless otherwise stated).** Total length 1.89–2.26, 2.11 mm. Wing length 0.98–1.11, 1.05 mm. Total length/wing length 1.90–2.15, 1.99. Wing length/length of profemur 2.21–2.53, 2.37.

***Colouration*.** Head, thorax, legs and abdomen uniformly brown. Wing membrane without dark markings.

***Antenna*. **AR = 0.32–0.39, 0.35. Thirteenth flagellomere 230–274, 260 µm long.

***Head*.** Temporal setae 7–8, 7 in single row. Clypeus with 10–14, 12 setae. Tentorium 47–75, 65 µm long, maximum width 12–20, 17 µm. Stipes 80–117, 100 (4) µm long. Palp segment lengths (in µm): 20–32, 27; 22–27, 25; 65–85, 72; 95–125, 110; 87–132, 115. Third palpomere with 2–5, 4 sensilla clavata subapically, longest 12–15, 14 µm long. Fifth palpomere/third palpomere 1.21–1.75, 1.54.

***Thorax*.** Dorsocentrals 6–8, 7 in single row, acrostichals 10–14, 12, prealars 2. Scutellum with 2 setae.

***Wing*.**VR = 1.21–1.32, 1.27. Brachiolum with 1 seta, R with 11–12, 11 setae, R_1_ with 5–8, 7 setae, R_4+5_ with 11–17, 15 setae, remaining veins bare.

***Legs*.** Spur of fore tibia 39–44, 42 µm long including 15–20, 17 µm long scale. Mid-tibia with 1 spur, 20–25, 22 µm long; hind tibia with 2 spurs, 20–25, 23 and 28–31, 30 µm long. Combs of mid-tibia 17–19, 18 µm long, of hind tibia 18–21, 19 µm long. Width at apex of fore tibia 29–39, 34 µm, of mid-tibia 34–39, 37 µm, of hind tibia 34–44, 39 µm. Lengths and proportions of legs as in Table 12.

**Table 12.** Lengths (in μm) and proportions of leg segments in *Nilothauma werekena* sp. nov., adult males (n = 5–7, unless otherwise stated).

	Fe		Ti		ta_1_	ta_2_
p_1_	374–501, 433		315–394, 345		443–522, 473	246–286, 266
p_2_	345–463, 424		296–345, 325		177–207, 197	89–99, 94
p_3_	443–532, 493		463–522, 502		266–305, 286	138–158, 148
	**ta_3_**		**ta_4_**		**ta_5_**	**LR**
p_1_	187–217, 207		118–148, 138 (4)		69–89, 79 (4)	1.36–1.47, 1.40
p_2_	69–79, 74		39–49, 44		30–39, 35	0.56–0.63, 0.60
p_3_	128–158, 148 (4)		89–99, 94 (4)		59–69, 55 (4)	0.57–0.61, 0.59
		**BV**		**SV**		**BR**
p_1_		1.84–2.20, 1.95		1.55–1.66, 1.60		2.0–3.3, 2.5
p_2_		3.50–4.25, 3.81		3.42–4.05, 3.71		2.3–4.7, 3.3
p_3_		2.70–2.89, 2.81 (4)		3.39–3.45, 3.41		5.0–7.0, 5.9

***Hypopygium*** (Fig. 14A–C). Tergite IX without dorsal lobe(s), with low, but wide median dorsal protruberance with 29–32, 31 strong, clustered median setae; posterior margin rounded to subrectangular, with 8–11, 9 weak setae to each side of base of anal point. Anal point spatulate, 37–47, 40 µm long, maximum width 5–10, 7 µm. Tergite bands not continuous. Laterosternite IX with 1–2, 1 seta; with anterolateral thorn. Phallapodeme 40–50, 45 µm long; transverse sternapodeme 15–25, 20 µm long. Gonocoxite 62–82, 72 µm long. Inferior volsella strongly curved, 22–30, 27 µm long, 5–8, 6 µm wide medially, with microtrichia and 3 strong, simple setae apically. Superior volsella tongue-shaped to slightly pediform, 32–40, 37 µm long, 15–25, 20 µm wide at base, densely covered with microtrichia. Median volsella fused to superior volsella, consisting of 2–3, 2 small tubercles, each bearing single, long seta. Gonostylus 70–100, 90 µm long, basal half curved, distal half straight. HR = 0.63–0.96, 0.83. HV = 2.26–2.70, 2.34.

##### Female adult and immatures.

Unknown.

##### Distribution

**(Fig. 16D).** Known from Barcelos (Amazonas State), in the Brazilian Amazon.

#### Nilothauma yekwana sp. nov.

Taxon classificationAnimaliaDipteraChironomidae

FCEDBDDB-900F-5620-8F46-CD06B44FCC0F

http://zoobank.org/2FDCF2C9-C8ED-4675-93F2-65B893098864

**Figure 15.**
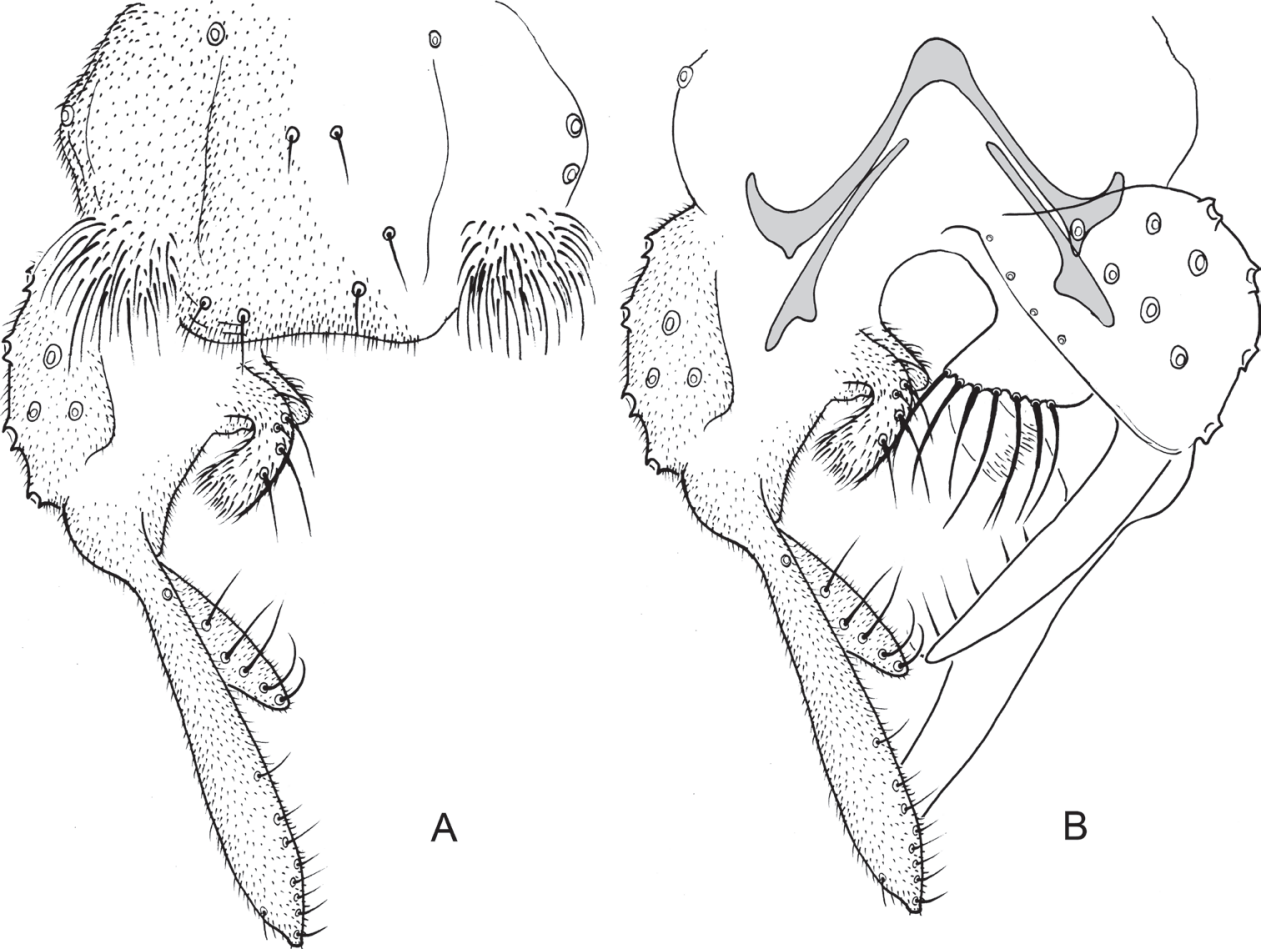
*Nilothauma yekwana* sp. nov. adult male **A** hypopygium, dorsal view **B** hypopygium with tergite IX removed, dorsal aspect to the left and ventral aspect to the right.

**Figure 16.**
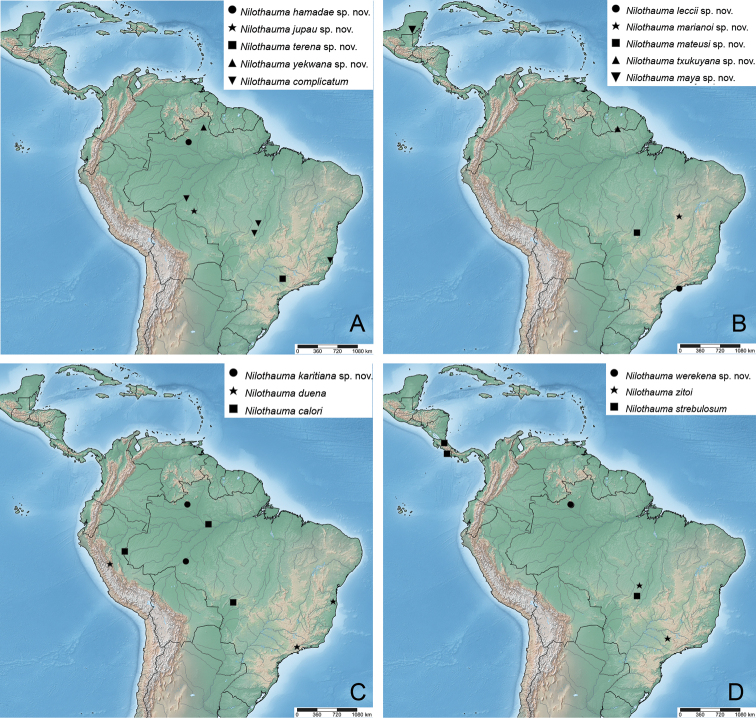
Distribution maps of Neotropical *Nilothauma* species **A***N. hamadae* sp. nov., *N. jupau* sp. nov., *N. terena* sp. nov., *N. yekwana* sp. nov., *N. complicatum* Mendes & Andersen, 2009 **B***N. leccii* sp. nov., *N. marianoi* sp. nov., *N. mateusi* sp. nov., *N. txukuyana* sp. nov., *N. maya* sp. nov. **C***N. karitiana* sp. nov., *N. duena* Roback, 1960, *N. calori* Mendes & Andersen, 2009 **D***N. werekena* sp. nov., *N. zitoi* Mendes & Andersen, 2009, *N. strebulosum* (Adam & Sæther, 2000).

Figures 15A, B , 16A

##### Type material.

***Holotype*** male, slide-mounted: Brazil, Roraima, Boa Vista, BR-174, Igarapé Água Boa, 02°43'32"N, 60°48'43"W, 2014, N. Hamada leg (UFSC).

##### Etymology.

The specific epithet honours the Ye’kwana, indigenous people from the Roraima State, Brazil. The name is to be regarded as a noun in apposition.

##### Diagnostic characters.

The male can be distinguished from its congeners by the combination of: tergite IX with two setose dorsolateral lobes; anal point absent; posterior margin of tergite IX subrectangular; inferior volsella straight, tapering to apex; superior volsella curved, projecting posteriolaterally; median volsella broad, triangular, bearing 7 strong setae.

##### Description.

**Male imago (n = 1).** Total length 1.77 mm. Wing length 0.91 mm. Total length/wing length 1.95. Wing length/length of profemur 2.19.

***Colouration*.** Head, thorax, legs and abdomen uniformly light brown. Wing membrane without dark markings.

***Antenna*.**AR = 0.22. Thirteenth flagellomere 137 µm long.

***Head*.** Temporal setae 7 in single row. Clypeus with 15 setae. Tentorium 65 µm long, maximum width 12 µm. Stipes 80 µm long. Palp segment I–III lengths (in µm): 22, 17, 50; segment IV and V lost. Third palpomere with 2 sensilla clavata subapically, longest 15 µm.

***Thorax*.** Dorsocentrals 5 in single row, acrostichals 10, prealars 2. Scutellum with 2 setae.

***Wing*.**VR = 1.48. Brachiolum with 1 seta, R with 6 setae, R_4+5_ with 2 setae at apex, remaining veins bare.

***Legs*.** Spur of fore tibia 44 µm long including 15 µm long scale. Mid-tibia with 1 spur, 20 µm long; hind tibia with 2 spurs, 20 and 25 µm long. Combs of mid-tibia 15 µm long, of hind tibia 18 µm long. Width at apex of fore tibia 39 µm, of mid-tibia 39 µm, of hind tibia 44 µm. Lengths and proportions of legs as in Table 13.

**Table 13.** Lengths (in μm) and proportions of leg segments in *Nilothauma yekwana* sp. nov., adult male (n = 1).

	Fe	Ti	ta_1_	ta_2_	ta_3_	ta_4_
p_1_	414	296	–	–	–	–
p_2_	394	256	177	59	39	30
p_3_	443	414	217	99	99	79
	**ta_5_**	**LR**	**BV**	**SV**	**BR**	
p_1_	–	–	–	–	–	–
p_2_	30	0.57	5.25	3.67	2.0	–
p_3_	49	0.52	3.60	3.95	4.4	–

***Hypopygium*** (Fig. 15A, B). Tergite IX with 2 dorsolateral, densely setose lobes, setae about 15 µm long; with 2 strong setae anterolaterally, 2 medially and 4 close to posterior margin; posterior margin subquadrangular, anal point absent. Tergite bands lacking. Laterosternite IX with 1 seta. Phallapodeme 37 µm long; transverse sternapodeme 10 µm long. Gonocoxite 65 µm long. Inferior volsella straight, tapering to apex, 40 µm long, 7 µm wide medially, with microtrichia and 9 simple setae subapically. Superior volsella curved, projecting posteriolaterally, 22 µm long, 5 µm wide at base, covered with microtrichia and fringed at apex. Median volsella broad, triangular, 15 µm long, with 7 strong setae (one of them bifid), longest 20 µm. Gonostylus 75 µm long, straight. HR = 0.87. HV = 2.36.

##### Female adult and immatures.

Unknown.

##### Distribution

**(Fig. 16A).** Known from Roraima State, Brazilian Amazon.

#### Nilothauma zitoi

Taxon classificationAnimaliaDipteraChironomidae

Mendes & Andersen, 2009

16C8DD1F-36C4-51F1-A3F9-665B38E359B0

Figure 16D

##### Additional material.

1 male, slide-mounted: Brazil, Mato Grosso, Ribeirão Cascalheira, Fazenda Campina Verde, Rio Suiá Miçu, 12°48.591'S, 52°06.925'W, 10.x.2007, light trap, L.C. Pinho, S. Mateus, L. Torati & F.R. Silva leg.

##### Distribution

**(Fig. 16D).** The species was originally described by Mendes and Andersen (2009), based on a single male from São Paulo State; the range is now extended to the Mato Grosso State.

**Figure 17.**
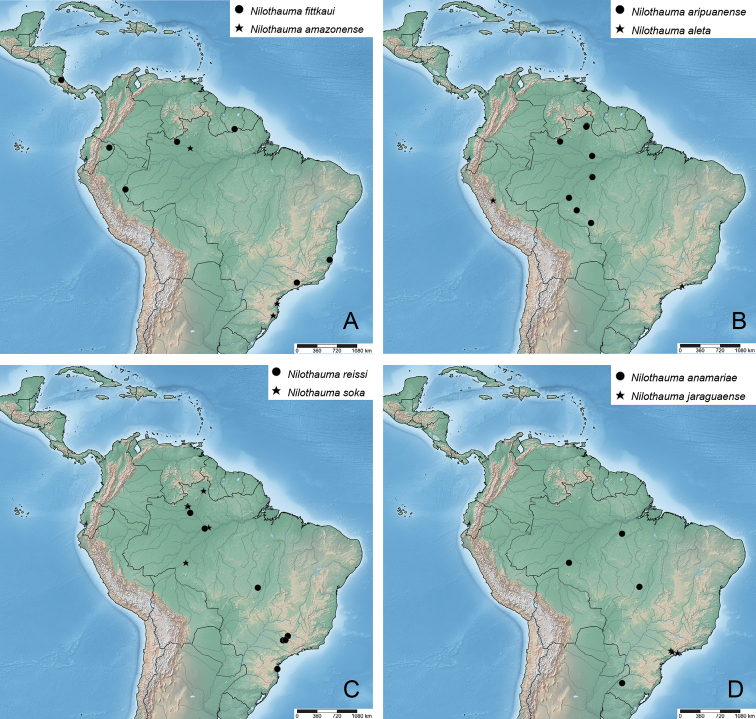
Distribution maps of Neotropical *Nilothauma* species **A***N. fittkaui* (Soponis, 1987), *N. amazonense* Mendes & Andersen, 2009 **B***N. aripuanense* Mendes & Andersen, 2009, *N aleta* Roback, 1960 **C***N. reissi* (Soponis, 1987), *N. soka* Andersen, Bello-González & Hagenlund, 2016 **D***N. anamariae* Dantas & Hamada, 2017, *N. jaraguaense* Mendes & Andersen, 2009.

#### Key to the males of *Nilothauma* Kieffer of the world

Modified from Qi et al. (2014), Niitsuma (2016) and Andersen et al. (2016), with the inclusion of sixteen species.

**Table:** 

1	Tergite IX without setose dorsal lobe(s) or projection(s)	**2**
–	Tergite IX with one to four setose dorsal lobes or projection(s) (e.g. Figs 16, 20)	**19**
2	Anal point present	**3**
–	Anal point absent	**15**
3	Tergite IX with median cluster of about 30 strong setae	**4**
–	Tergite IX with few, clustered setae, if numerous they are scattered (as in *N. aripuanense*)	**5**
4	Superior volsella slender, without microtrichia and with lateral spine. Brazil	***Nilothauma jaquei* Dantas & Hamada, 2017**
–	Superior volsella pediform to lingulate, covered with microtrichia and without lateral spine (Fig. 14). Brazil	***Nilothauma werekena* sp. nov.**
5	Wing with conspicuous dark markings (Fig. 5); abdominal tergites II, III, and VI–VIII dark brown. Brazil	***Nilothauma jupau* sp. nov.**
–	Wing unmarked, at most with faint colour (as in *Nilothauma aleta*, best seen in dark-field filter); abdominal tergites uniformly pale to brown	**6**
6	Gonostylus stout or swollen (Figs 1, 3, 6, 7)	**7**
–	Gonostylus slender (Fig. 10)	**10**
7	Gonostylus very long, narrow basally and apically, swollen at mid-length (Fig. 6). Brazil	***Nilothauma karitiana* sp. nov.**
–	Gonostylus stout, not distinctly swollen at mid-length (Figs 1, 3)	**8**
8	Acrostichals absent; anal point wide, covering most setae along posterior margin of tergite IX (Fig. 1). Peru, Brazil	***Nilothauma aleta* Roback, 1960**
–	Acrostichals present; anal point comparatively narrow, nearly parallel-sided, with most setae placed lateral to base of anal point	**9**
9	Superior volsella tapering to apex; inferior volsella short, stout, with short, simple setae (Fig. 3). Peru, Brazil	***Nilothauma duena* Roback, 1960**
–	Superior volsella wider at mid-length; inferior volsella long and slender, with long simple or apically split setae (Fig. 7). Brazil	***Nilothauma leccii* sp. nov.**
10	Superior volsella narrow, straight, curved or weakly sinuous, projecting posterio-medially, with one to six apical setae	**11**
–	Superior volsella wider in distal half, projecting posterio-medially or posterio-laterally, with microtrichia only	**13**
11	Tergite IX with numerous scattered setae; anal point broadly lanceolate, about 20 µm wide. Brazil	***Nilothauma aripuanense* Mendes & Andersen, 2009**
–	Tergite IX with one to four median setae and about 12 setae along posterior margin; anal point comparatively narrow	**12**
12	Tergite IX with single seta anterior to anal point; gonostylus with two to four sub-basal dorsal setae not arising from protuberances. Brazil	***Nilothauma paucisetis* Dantas & Hamada, 2017**
–	Tergite IX with four aligned setae anterior to anal point; gonostylus with single sub-basal seta arising from distinct inner protuberance (Fig. 10). Neotropical Mexico	***Nilothauma maya* sp. nov.**
13	Anal point parallel-sided, about 4 µm wide; inferior volsella with about 18 slender, simple setae (Fig. 8). Brazil	***Nilothauma marianoi* sp. nov.**
–	Anal point spatulate, 15–23 µm wide; inferior volsella with few (about 3) simple, slender setae or numerous (about 12) stout, split setae apically	**14**
14	Superior volsella boot-shaped, projecting posterior-laterally; inferior volsella narrow, with few simple, slender setae apically. Brazil	***Nilothauma soka* Andersen, Bello-González & Hagenlund, 2016**
–	Superior volsella straight, projecting posterior-medially; inferior volsella wide, with numerous stout, split setae apically. Brazil	***Nilothauma anamariae* Dantas & Hamada, 2017**
15	Inferior volsella branched subapically. Brazil	***Nilothauma complicatum* Mendes & Andersen, 2009**
–	Inferior volsella simple	**16**
16	Superior volsella pediform, without ventral transverse fold, with setae and microtrichia	**17**
–	Superior volsella diamond-shaped, with ventral transverse fold, with microtrichia only	**18**
17	Wing vein R_1_ with setae; gonostylus nearly parallel-sided in apical half. Brazil, Ecuador	***Nilothauma fittkaui* (Soponis, 1987)**
–	Wing vein R_1_ bare; gonostylus widest in apical one-third. Brazil	***Nilothauma reissi* (Soponis, 1987)**
18	Apex of superior volsella projecting caudad. Brazil	***Nilothauma sooretamense* Mendes & Andersen, 2009**
–	Apex of superior volsella projecting mesad. Brazil	***Nilothauma involucrum* Mendes & Andersen, 2009**
19	Anal point lacking or rudimentary, completely covered by microtrichia	**20**
–	Anal point present, with microtrichia at most in basal half	**22**
20	Tergite IX with four lobes or projections, one anterior pair with strong, long setae and one posterio-lateral, triangular pair with weaker setae	**21**
–	Tergite IX with one or two dorsal lobes	**27**
21	Tergite IX with posterio-lateral pair of projections short, subequal in length to anterior pair (Fig. 11); laterosternite IX with thorn. Brazil	***N. terena* sp. nov.**
–	Tergite IX with posterio-lateral pair of projections long, more than three times longer than anterior pair (Fig. 13); laterosternite IX without thorn. Brazil	***N. txukuyana* sp. nov.**
22	Dorsal projections of tergite IX differ in shape. Ghana	***Nilothauma insolitum* Adam & Sæther, 1999**
–	Dorsal projections of tergite IX of the same shape	**23**
23	Median volsella fused to superior volsella; superior volsella broadly pediform. Brazil	***Nilothauma fazzariense* Mendes & Andersen, 2009**
–	Median volsella distinct and separated from superior volsella; superior volsella digitate, curved, with or without lateral spine	**24**
24	Dorso-lateral projections of tergite IX overreaching posterior margin of tergite. Brazil	***Nilothauma roquei* Mendes & Andersen, 2009**
–	Dorso-lateral projections of tergite IX not extended beyond posterior margin of tergite	**25**
25	Superior volsella with lateral spine; laterosternite IX with thorn; posterior margin of tergite IX broadly rounded. Brazil	***Nilothauma calori* Mendes & Andersen, 2009**
–	Superior volsella without lateral spine; laterosternite IX without thorn; posterior margin of tergite IX subrectangular	**26**
26	Median volsella consisting of single, small tubercle bearing one seta; inferior volsella curved, not tapering to apex. Brazil, Costa Rica	***Nilothauma strebulosum* (Adam & Sæther, 2000)**
–	Median volsella broad, triangular, bearing 7 strong setae; inferior volsella straight, tapering to apex (Fig. 15). Brazil	***Nilothauma yekwana* sp. nov.**
27	Tergite IX with single, median setose dorsal lobe	**28**
–	Tergite IX with two setose dorsal lobes	**37**
28	Superior volsella without microtrichia	**29**
–	Superior volsella covered with microtrichia	**32**
29	Dorsal projection weakly developed and undivided; situated posteriorly on tergite IX, close to base of anal point. China, Japan, Thailand	***Nilothauma japonicum* Niitsuma, 1985**
–	Dorsal projection well developed, divided or undivided, situated anteriorly on tergite IX, at some distance from base of anal point	**30**
30	Dorsal projection three-pronged at apex, without setae. Ghana	***Nilothauma fuscina* Adam & Sæther, 1999**
–	Dorsal projection bell-shaped, with numerous setae	**31**
31	Superior volsella with single apical seta; anal point narrow. Brazil	***Nilothauma matogrossense* Mendes & Andersen, 2009**
–	Superior volsella with one dorsolateral and one apical spine-like seta; anal point broad. Ghana	***Nilothauma duminola* Adam & Sæther, 1999**
32	Anal point very broad, about half as wide as tergite IX and lanceolate (Fig. 4). Brazil	***Nilothauma hamadae* sp. nov.**
–	Anal point narrow, spatulate to tapering, but not lanceolate	**33**
33	Anal point tapering or only slightly widened medially	**34**
–	Anal point distinctly spatulate	**35**
34	Wing with distinct dark areas at RM, FCu, along apical half of An and in cells r_4+5_ and m_1+2_. Cuba	***Nilothauma granma* Andersen, Bello-González & Hagenlund, 2016**
–	Wing without dark areas. Canada, USA	***Nilothauma babiyi* (Rempel, 1937)**
35	Dorsal projection large, covering most of tergite IX. Australia	***Nilothauma adunatum* Adam & Sæther, 1999**
–	Dorsal projection of tergite IX small, with setae at apex only	**36**
36	Wing length > 2.4 mm; AR = 0.28; median volsella consisting of two tubercles, each with single, strong apical seta. Canada	***Nilothauma verrucum* Adam & Sæther, 1999**
–	Wing length < 1.3 mm; AR = 0.13; median volsella consisting of three tubercles, each with strong, apical seta. Venezuela	***Nilothauma canaima* Andersen, Bello-González & Hagenlund, 2016**
37	Dorsal projections of tergite IX of the same shape	**38**
–	Dorsal projections of tergite IX of different shapes	**42**
38	Anal point short, digitiform, with microtrichia in basal half; gonostylus distinctly widened in apical one-third. Brazil	***Nilothauma zitoi* Mendes & Andersen, 2009**
–	Anal point well developed, lanceolate or parallel-sided; gonostylus nearly parallel-sided in apical half	**39**
39	Superior volsella short, subtriangular, with two apical tubercles bearing setae; inferior volsella with long, strong simple setae (Fig. 9). Brazil	***Nilothauma mateusi* sp. nov.**
–	Superior volsella long, pediform to tongue-shaped; inferior volsella with slender, simple apically split setae	**40**
40	Anal point parallel-sided; laterosternite IX with thorn. Chile	***Nilothauma spiesi* Mendes & Andersen, 2009**
–	Anal point lanceolate; laterosternite IX without thorn	**41**
41	Inferior volsella and gonostylus with apically split setae; median volsella curved, tapering, with microtrichia and setae. Brazil	***Nilothauma jaraguaense* Mendes & Andersen, 2009**
–	Inferior volsella and gonostylus with simple setae only; median volsella short, parallel-sided, with two apical setae, without microtrichia. Brazil	***Nilothauma amazonense* Mendes & Andersen, 2009**
42	Wing with dark areas or bands; anterior projection on tergite IX long and deeply divided	**43**
–	Wing without dark markings; anterior projection on tergite IX variably developed	**50**
43	Anterior projection on tergite IX with setae not restricted to apex. South Africa	***Nilothauma harrisoni* Adam & Sæther, 1999**
–	Anterior projection on tergite IX with apical setae only	**44**
44	Posterior projection on tergite IX deeply divided into antero-dorsal and postero-ventral parts	**45**
–	Posterior projection on tergite IX not as above	**47**
45	Anterior projection on tergite IX with simple, separated, apical setae; anterior part of posterior projection apically pointed; wing with postero-median spot extending on both sides of Cu_1_. Afrotropical	***Nilothauma pictipenne* Kieffer, 1921**
–	Anterior projection on tergite IX with setae forming fan-like structure; anterior part of posterior projection with blunt apex; wing with postero-median spot exclusively proximal of Cu_1_	**46**
46	Wing with four dark areas; setae on anterior projection on tergite IX branched apically. Ghana	***Nilothauma flabellatum* Adam & Sæther, 1999**
–	Wing with three dark areas; apical setae on anterior projection on tergite IX lamellate, not branched. Ghana	***Nilothauma kakumense* Adam & Sæther, 1999**
47	Posterior projection on tergite IX either with a disto-dorsal lobe or subapical constriction	**48**
–	Posterior projection on tergite IX without distal lobe or constriction	**49**
48	Posterior projection on tergite IX with long antero-lateral arms and disto-dorsal lobe. Tanzania	***Nilothauma anderseni* Adam & Sæther, 1999**
–	Posterior projection on tergite IX without antero-lateral arms, with apical constriction and five apical setae. Zimbabwe	***Nilothauma latocaudatum* Adam & Sæther, 1999**
49	Posterior projection on tergite IX without microtrichia, except on long, antero-lateral arms; superior volsella without antero-median extension; median volsella present. China, Japan	***Nilothauma nojirimaculatum* Sasa, 1991**
–	Posterior projection on tergite IX without antero-lateral arms, covered with microtrichia; superior volsella with antero-median extension; median volsella apparently absent. Japan	***Nilothauma hibaraquartum* Sasa, 1993**
50	Anterior projection on tergite IX with apically plumose setae	**51**
–	Apical setae on anterior projection on tergite IX not plumose	**58**
51	Anal point with microtrichia along median ridge and apical margin. Oriental China	***Nilothauma pandum* Qi, Lin, Wang & Shao, 2014**
–	Anal point without microtrichia	**52**
52	Superior volsella pad-like, with extensive microtrichia. Palaearctic Japan	***Nilothauma hibaratertium* Sasa, 1993**
–	Superior volsella slender, microtrichia absent or limited within small area when present	**53**
53	Superior volsella without lateral spur. Thailand	***Nilothauma mergae* Adam & Sæther, 1999**
–	Superior volsella with lateral spur	**54**
54	Superior volsella without setal brush or fringe, with only one to three apical setae	**55**
–	Superior volsella with apical setal brush or fringe	**56**
55	Anal point with microtrichia along median ridge; superior volsella relatively long when compared to median volsella (length ratio, Svo/Mvo > 4.0); inferior volsella with simple setae only. Oriental China	***Nilothauma aristatum* Qi, Tang & Wang, 2016**
–	Anal point bare; length ratio Svo/Mvo about 2.0; inferior volsella with apically split setae. Oriental China	***Nilothauma acre* Adam & Sæther, 1999**
56	Superior volsella four to five times as long as median volsella. Palaearctic Japan	***Nilothauma niidaensis* Niitsuma, 2016**
–	Superior volsella two to three times as long as median volsella	**57**
57	Anterior projection on tergite IX two to four times as long as broad. Canada, USA	***Nilothauma bicorne* (Townes, 1945)**
–	Anterior projection on tergite IX broader than long. USA	***Nilothauma mirabile* (Townes, 1945)**
58	Anal point trifid; anterior projection on tergite IX very long, tapering to parallel-sided apex, with apical setae only; posterior projection on tergite IX distally very slender, with five apical setae. D. R. Congo, Ghana	***Nilothauma burmeisteri* Adam & Sæther, 1999**
–	Anal point simple; anterior projection on tergite IX wart-like, with setae not only at apex; posterior projection on tergite IX triangular or apically rounded	**59**
59	Posterior projection on tergite IX apically rounded; superior volsella with two to four lobes	**60**
–	Posterior projection on tergite IX triangular; superior volsella without lobes	**61**
60	Anterior projection on tergite IX partially divided apically; superior volsella with two lobes. Oriental China	***Nilothauma bilobatum* Qi, Tang & Wang, 2016**
–	Anterior projection on tergite IX undivided apically; superior volsella with four lobes. Oriental China	***Nilothauma quatuorlobum* Yan, Tang & Wang, 2005**
61	Anal point parallel-sided; anterior projection on tergite IX with setae thickened at apices. Ghana	***Nilothauma ankasense* Adam & Sæther, 1999**
–	Anal point spatulate; anterior projection on tergite IX with setae not thickened at apices	**62**
62	Superior volsella tapering, widest near base. Europe	***Nilothauma brayi* (Goetghebuer, 1921)**
–	Superior volsella widest about one-third from apex. Australia	***Nilothauma infissum* Adam & Sæther, 1999**

## Supplementary Material

XML Treatment for Beardius longissimus

XML Treatment for Nilothauma

XML Treatment for Nilothauma aleta

XML Treatment for Nilothauma amazonense

XML Treatment for Nilothauma anamariae

XML Treatment for Nilothauma aripuanense

XML Treatment for Nilothauma calori

XML Treatment for Nilothauma complicatum

XML Treatment for Nilothauma duena

XML Treatment for Nilothauma fittkaui

XML Treatment for Nilothauma hamadae

XML Treatment for Nilothauma jaraguaense

XML Treatment for Nilothauma jupau

XML Treatment for Nilothauma karitiana

XML Treatment for Nilothauma leccii

XML Treatment for Nilothauma marianoi

XML Treatment for Nilothauma mateusi

XML Treatment for Nilothauma maya

XML Treatment for Nilothauma reissi

XML Treatment for Nilothauma soka

XML Treatment for Nilothauma strebulosum

XML Treatment for Nilothauma terena

XML Treatment for Nilothauma txukuyana

XML Treatment for Nilothauma werekena

XML Treatment for Nilothauma yekwana

XML Treatment for Nilothauma zitoi
